# Supplementary material for: Haploid genetic screens identify SPRING/C12ORF49 as a determinant of SREBP signaling and cholesterol metabolism
Source: Nat Commun. 2020 Feb 28;11:1128. doi: 10.1038/s41467-020-14811-1 (PMC7048761; doi:10.1038/s41467-020-14811-1)
Supplement: Supplementary file 1 — Supplementary Information [file 41467_2020_14811_MOESM1_ESM.pdf]

Supplementary information

Haploid genetic screens identify SPRING/C12ORF49 as a determinant of SREBP signaling and cholesterol metabolism

Loregger *et al.*

Supplementary Figure 1

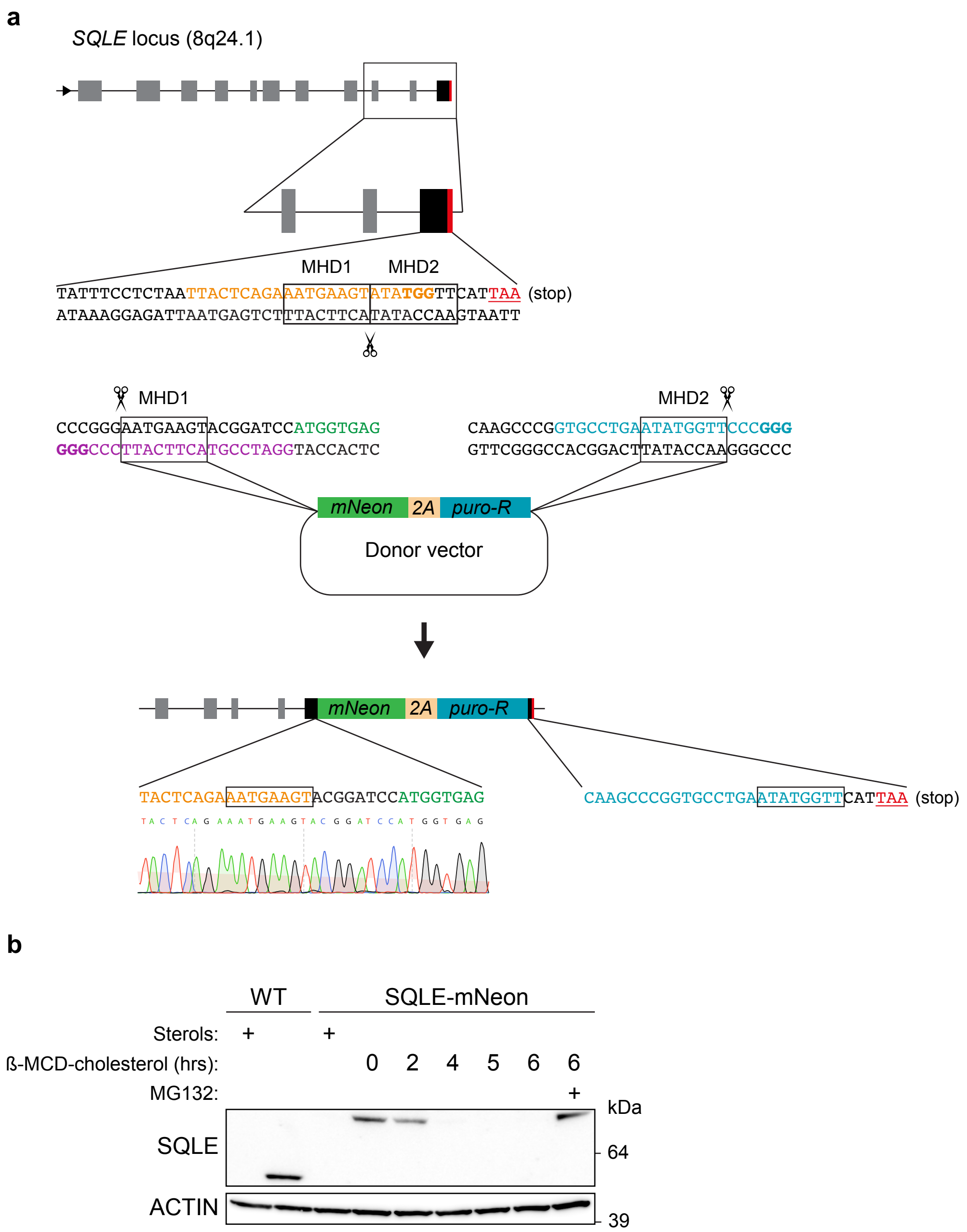

Supplementary Figure 1. Generation of Hap1 SQLE-mNeon cells. (*A*) Schematic illustration of CRISPR/Cas9-mediated targeting of the endogenous *SQLE* locus for in-frame integration of mNeon-2A-PURO. The 5' and 3' microhomology domains MHD1 and MHD2, respectively are boxed. The sgRNA target site in the endogenous *SQLE* locus is shown in orange, and the ones in the donor plasmid in purple and blue, with the respective PAM sites highlighted in bold. The correct integration of the donor cassette was verified by Sanger sequencing of amplified genomic DNA. (***B***) Hap1-WT and Hap1-SQLE-mNeon cells were cultured for 24 hrs in the presence or absence of sterols. Subsequently, 50 µg/ml β-methyl-cyclodextrin-cholesterol was added with or without 25 µM MG132 to inhibit the proteasome for the indicated time (N=3). Total cell lysates were immunoblotted as shown.

Supplementary Figure 2

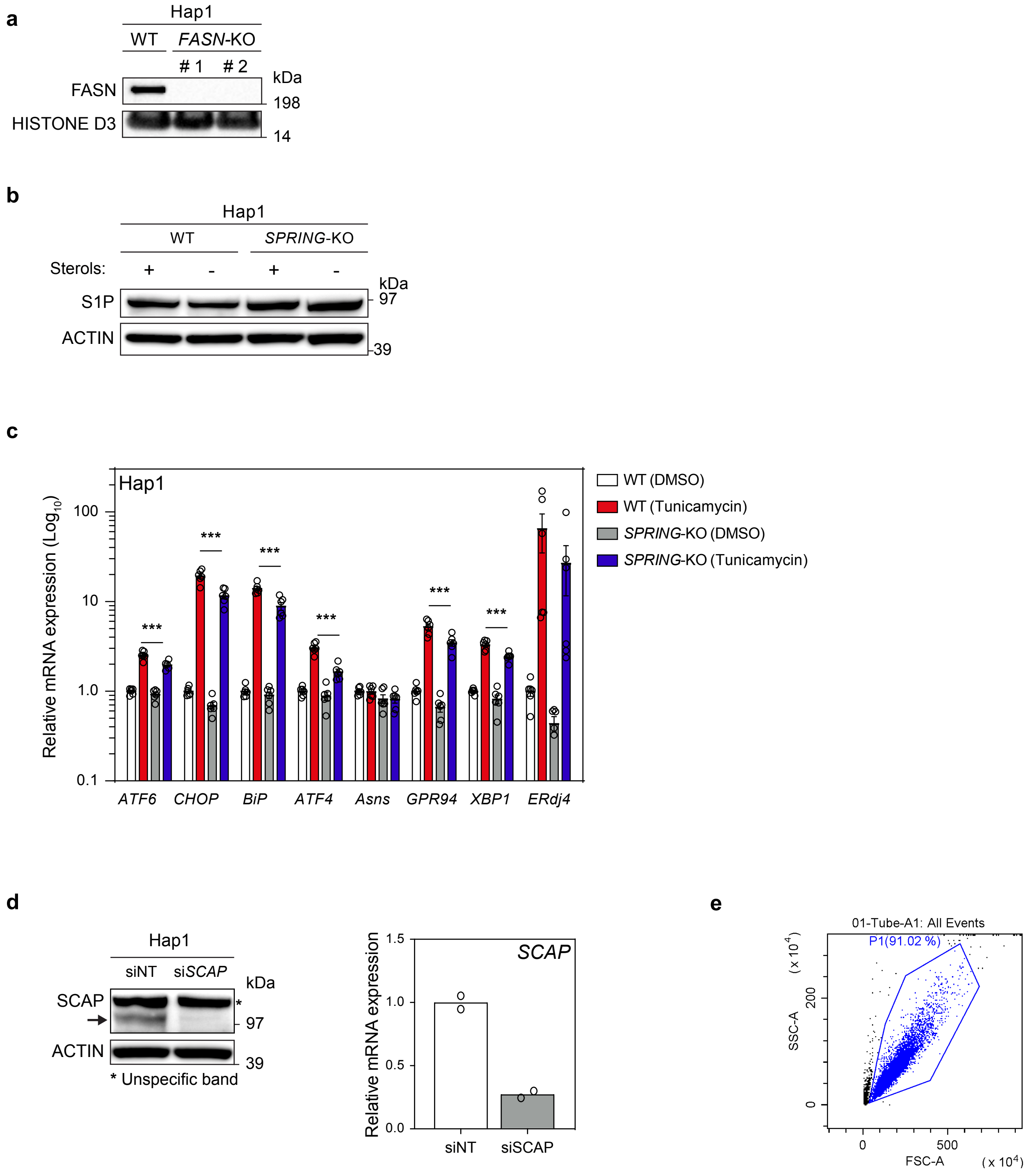

Supplementary Figure 2. (***A***) **Absence of FASN in Hap1-FASN^{KO} clones.** Total cell lysates of Hap1-WT and 2 independent Hap1-FASN^{KO} clones were immunoblotted as indicated. (***B***) **S1P levels are unchanged in cells lacking *SPRING*.** Hap1-WT and Hap1-SPRING^{KO} cells were cultured in the presence or absence of sterols and total cell lysates from Hap1-WT and Hap1-SPRING^{KO} cells were immunoblotted as indicated. (***A,B***) Representative images of two independent experiments are shown. (***C***) **Attenuated response to Tunicamycin in cells lacking *SPRING*.** Hap1-WT and Hap1-SPRING^{KO} cells were cultured in the presence or absence of 2 µg/mL Tunicamycin for 8 hrs. Subsequently, total RNA was isolated and expression of the indicated genes was determined by qPCR (N=6 biologically independent samples). Each bar and error represent the mean ± SEM, *** $p < 0.001$. Note that the Y-axis is in logarithmic scale. (***D***) **Validation of SCAP antibody.** Hap1 cells were transfected with siNT (Non-Targeting control) or siSCAP for 72 hrs. Subsequently, cells were harvested for (***left***) immunoblotting and (***right***) gene expression analysis as indicated (N=2 biologically independent samples). (***E***) **Generic FACS gating strategy.** A standard FSC vs. SSC gating strategy was used to exclude cell debris.

Supplementary Figure 3

a

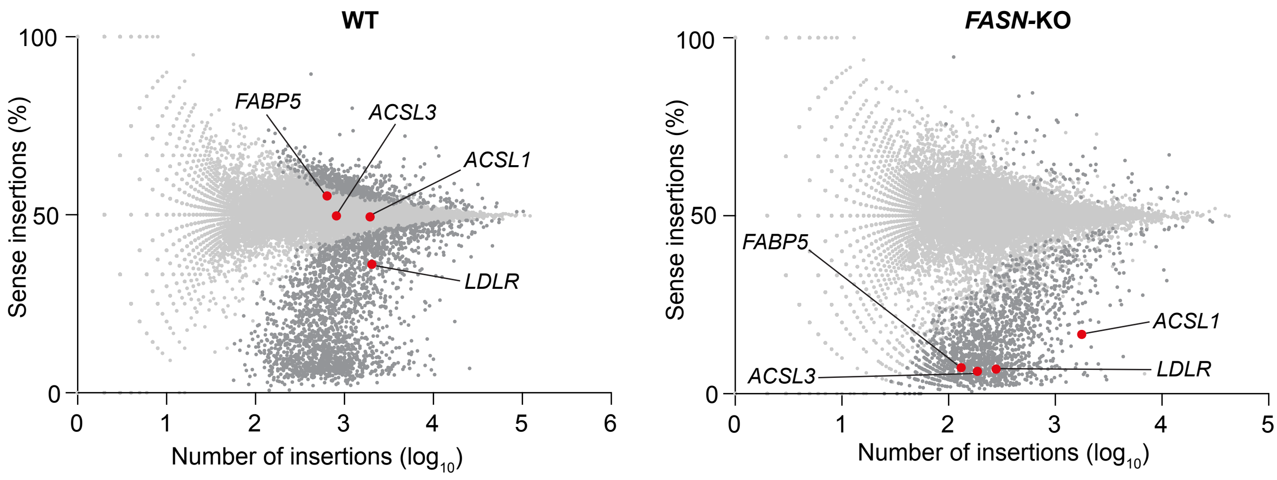

b

KO / WT > 2

Gene onthology (GO) gene set name	#Genes in gene set (K)	#Genes in overlap(k)	k/K	p-value	FDR q-value
GO_ DNA binding transcription factor activity	1701	59	0.0347	1.64E-18	2.5E-14
GO_ Biological adhesion	1417	43	0.0303	9.35E-12	7.15E-8
GO_ cell/cell adhesion via plasma membrane / adhesion molecules	274	19	0.0693	1.42E-11	7.23E-8
GO_ Intrinsic component of plasma membrane	1708	45	0.0263	2.94E-10	1.13E-6
GO_ Homophilic cell adhesion via plasma membrane / adhesion molecules	168	14	0.0833	6.50E-10	1.99E-6
GO_ cell/cell adhesion	827	27	0.0326	1.90E-8	4.84E-5
GO_ calcium/ion binding	698	23	0.0330	1.87E-7	4.10E-4
GO_ Tube development	1105	28	0.0253	1.75E-6	3.08E-3
GO_ Anatomical structure formation involved in morphogenesis	1172	29	0.0247	1.81E-6	3.08E-3
GO_ Second messenger mediated signalling	442	16	0.0362	4.35E-6	6.66E-3

WT / KO > 2

Gene onthology (GO) gene set name	#Genes in gene set (K)	#Genes in overlap(k)	k/K	p-value	FDR q-value
GO_Intrinsic component of plasma membrane	1708	102	0.0597	2.97E-19	4.55E-15
GO_ Neurogenesis	1599	88	0.0550	1.27E-14	9.71E-11
GO_ Regulation of transport	1842	95	0.0516	5.13E-14	2.61E-10
GO_ Neuron differentiation	1348	77	0.0571	9.96E-14	3.81E-10
GO_ Neuron part	1715	89	0.0519	2.47E-13	7.55E-10
GO_ Plasma membrane region	1168	70	0.0590	3.06E-13	7.80E-10
GO_ Ion transport	1677	86	0.0513	1.22E-12	2.66E-9
GO_ ion transmembrane transport	1138	66	0.0580	3.27E-12	6.26E-9
GO_ synapse	1171	67	0.0572	4.06E-12	6.90E-9
GO_ regulation of membrane potential	443	37	0.0855	5.60E-12	8.57E-9

Supplementary Figure 3. (*A*) **Various genes that are essential in Hap1-FASN^{KO} cells are related to lipid import.** Comparison of gene-essentiality screens between Hap1-WT cells and Hap1-FASN^{KO} cells. Per gene, the ratio of sense/total orientation gene-trap insertions (*y* axis) and the total number of insertions in a particular gene (*x* axis) are plotted. FASN synthetic lethality screens shows that a set of lipid metabolism-associated genes is depleted in FASN^{KO} cells. (*B*) **Gene onthology analysis of differentially expressed genes.** Genes that displayed 2-fold change between log_2 normalized expression values (higher or lower) between Hap1-WT and Hap1-SPRING^{KO} cells were subjected to Gene Onthology (GO) analysis. The top 10 GO emerging pathways are indicated.

Supplementary Figure 4

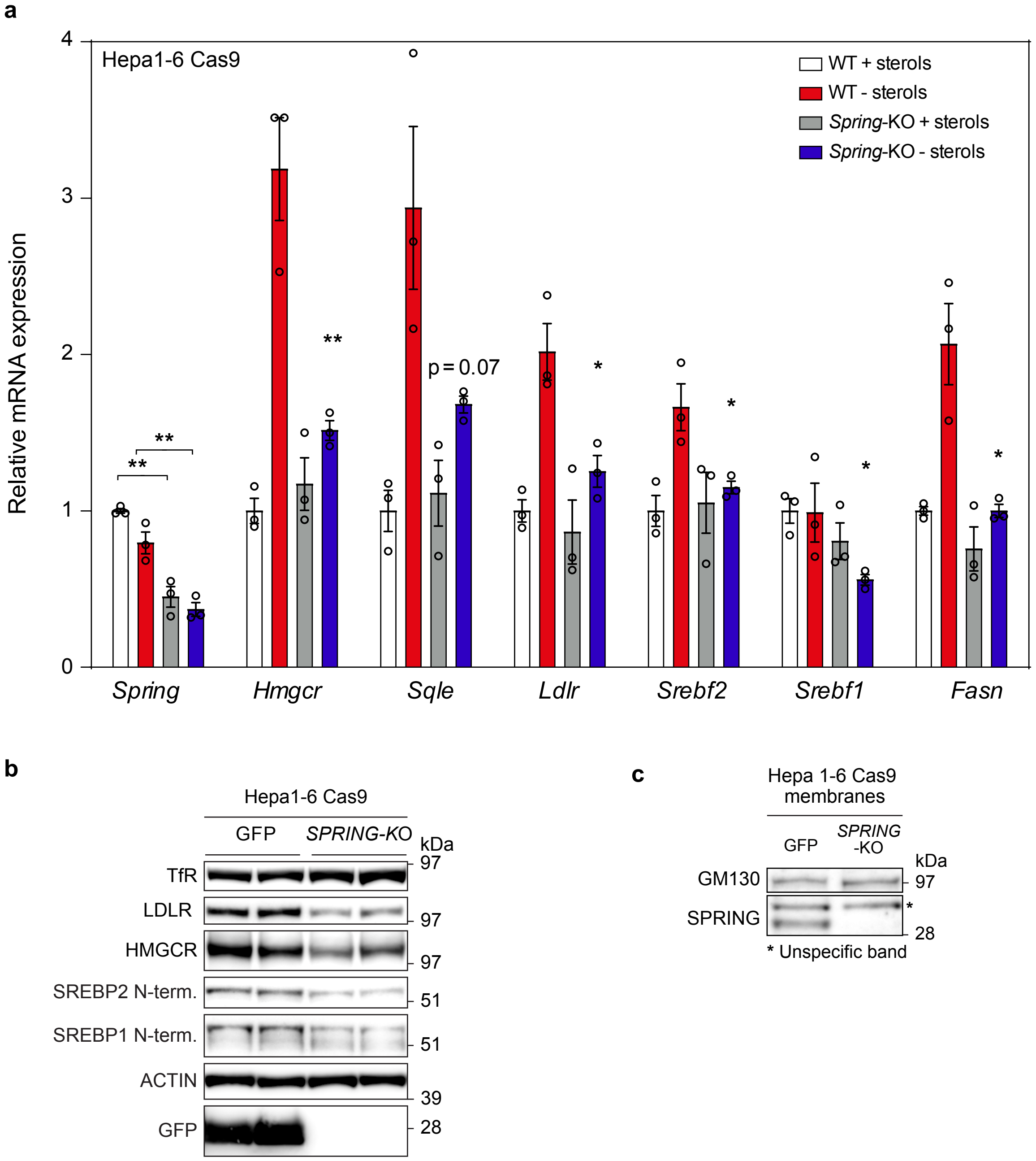

Supplementary Figure 4. Loss of Spring in murine Hepa1-6-Cas9 hepatoma cells attenuates activity of the SREBP pathway. Hepa1-6-Cas9 cells were infected with Ad-3x-sgRNA-Spring or Ad-GFP adenoviral particles at an MOI of 50 for 72 hrs. Subsequently, cells were cultured in the presence or absence of sterols. Total RNA was isolated and expression of the indicated genes determined by qPCR (N=3 biologically independent samples). Each bar and error represent the mean ± SEM, * $p < 0.05$, ** $p < 0.01$. (***B,C***) Cells were treated as in (***A***) and total cell lysates or crude membrane preparations were immunoblotted as indicated (N=2-4).

Supplementary Figure 5

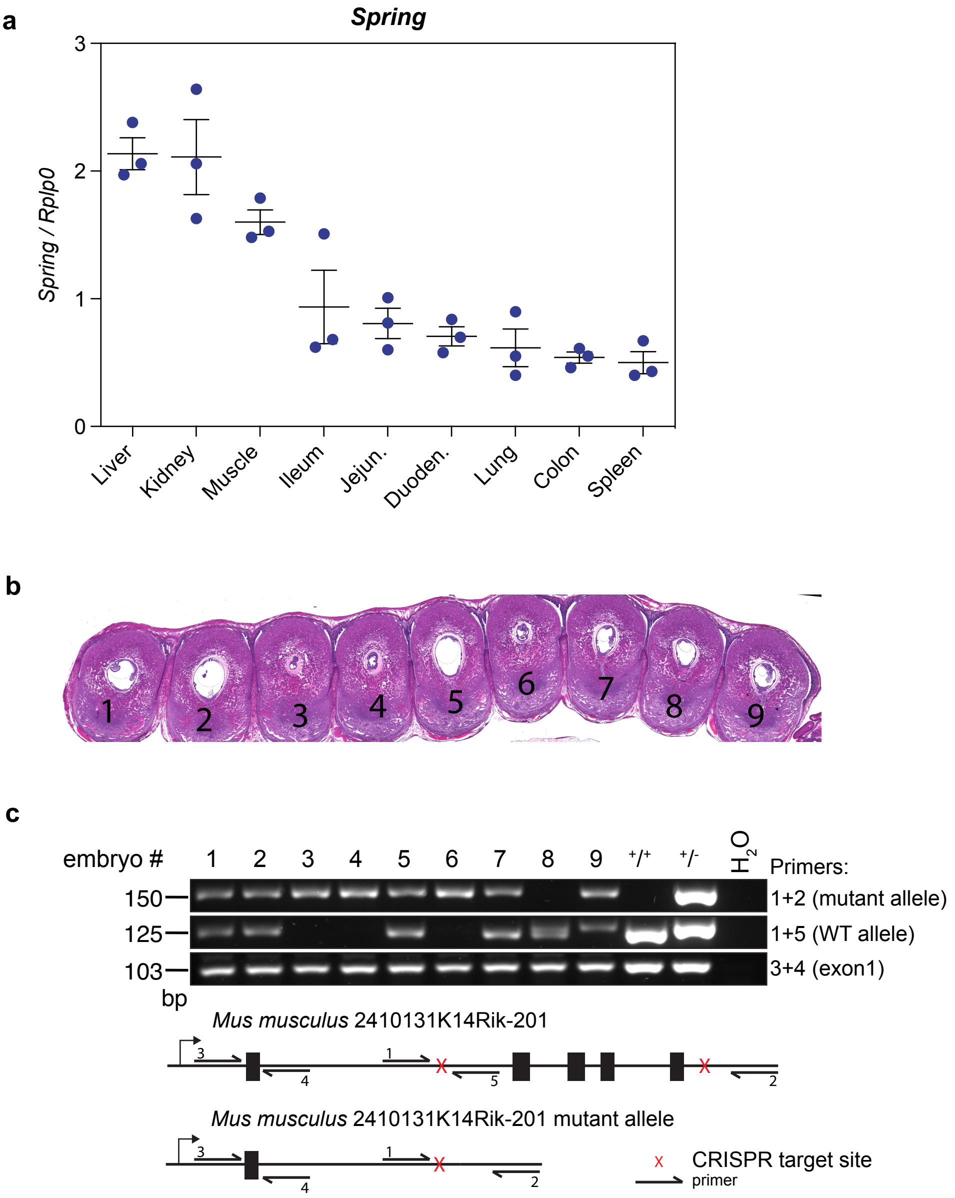

Supplementary Figure 5. Homozygous loss of Spring is embryonic lethal. (*A*) The indicated tissues were collected from male C57Bl/6J mice and the expression of *Spring* was determined by qPCR. Each point represents an individual animal and the mean ± SD is plotted. (N=3 animals/group) (***B***) Mouse uterine horn showing 9 embryos at the 7.5 dpc stage. Embryos # 3, 4, and 6 were smaller and had a poorly developed amniotic cavity and allantois in comparison with the adjacent normally developed embryos. (***C***) Using laser microdissection, tissue from each single embryo was obtained and genomic DNA was isolated. Each lane represents an independent embryo. PCR analysis showed that the WT allele was absent in poorly-developed embryos (#3, 4 and 6). The location of the oligonucleotides used for PCR amplification are shown.

Supplementary Figure 6

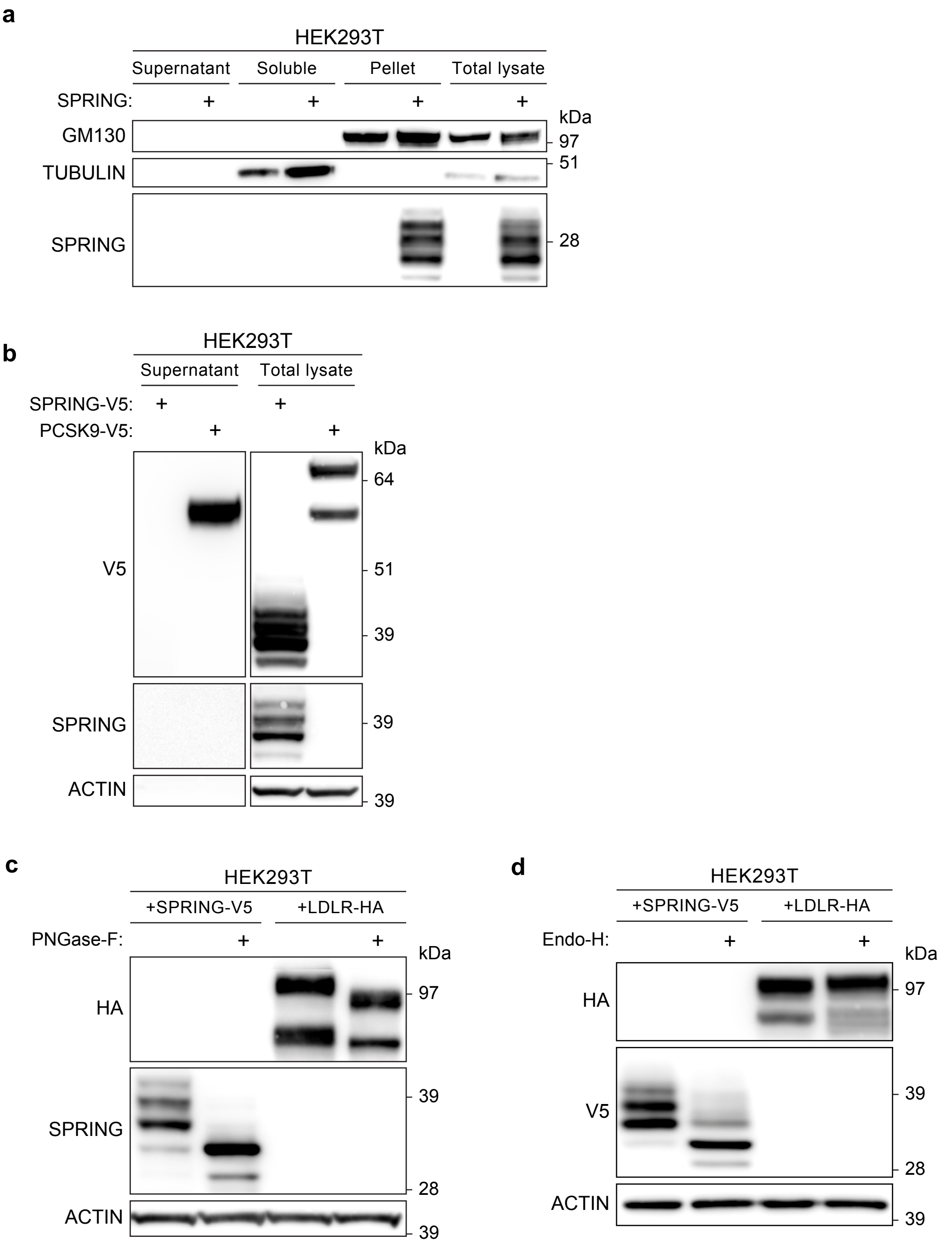

Supplementary Figure 6. SPRING is a highly glycosylated membrane protein. (***A***) HEK293T cells were transiently transfected with a SPRING expression construct for 48 hrs, after which the medium was collected and total cell lysates were collected and fractionated. Samples from the differently collected fractions and from the total cell lysates were immunoblotted as indicated (N=3). Tubulin and GM130 were used to discriminate between the soluble and pellet fractions. (***B***) HEK293T cells were transiently transfected with a SPRING-V5 or PCSK9-V5 expression constructs. Total cell lysates and culture supernatants were collected and immunoblotted as indicated. Note that PCSK9, a secreted protein, is readily identified in the supernatant while SPRING is not. (***C***) HEK293T cells were transiently transfected with SPRING or LDLR expression constructs. Total cell lysates were incubated with PNGase-F for 3 hrs and subsequently immunoblotted as indicated. (***D***) Cells were treated as in (***C***) and subjected to EndoH digestion for 1 hr. Note that the LDLR is known to undergo glycosylation, which is sensitive to PNGase-F activity, but not to EndoH activity. (***B,C,D***) Representative images of three independent experiments are shown.

Supplementary Figure 7

a

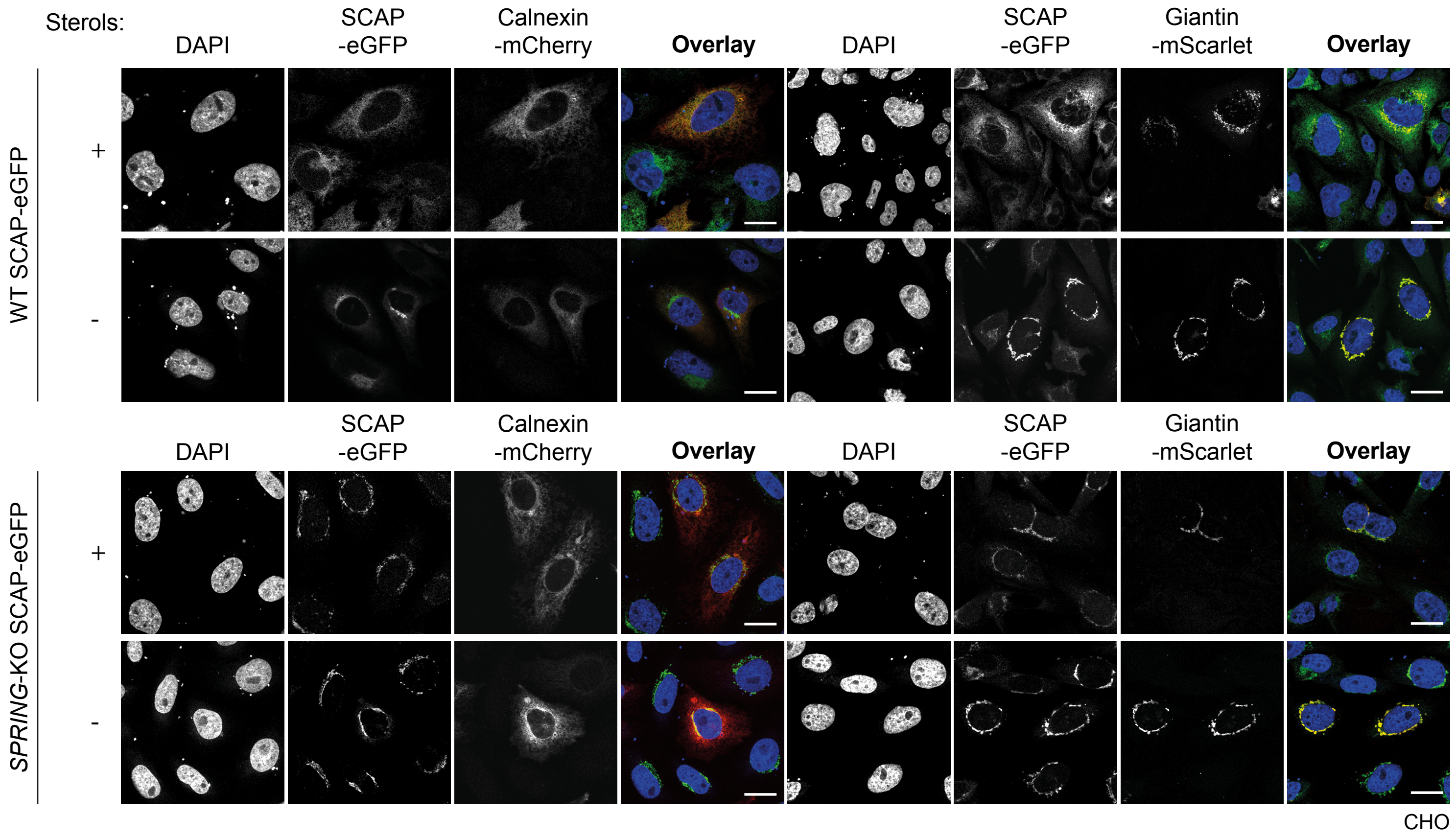

b

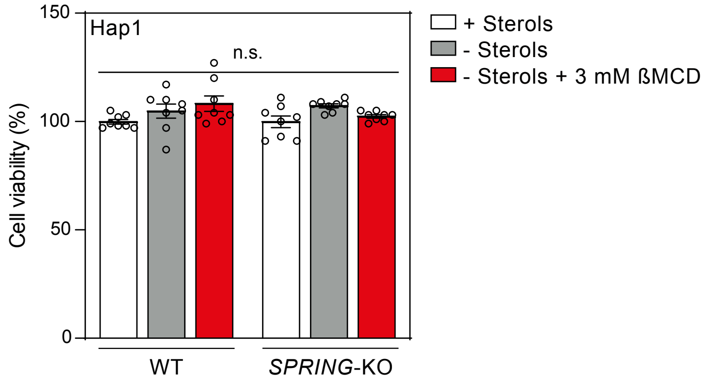

Supplementary Figure 7. Supplementary results accompanying main Figure 6. (*A*) CHO-SCAP-eGFP-WT and CHO-SCAP-eGFP-SPRING^{KO} were transfected with expression plasmids encoding Calnexin-mCherry (proxy for ER) or Giantin-mScarlet (proxy for Golgi). Cells were cultured in the presence or absence of sterols for 16 hrs before imaging. Representative fluorescence images are shown; scale bar, 10 µm. Note that in SCAP-eGFP-WT cells, in the presence of sterols SCAP is largely localized in the ER together with Calnexin, and shifts towards colocalization with Giantin in the Golgi upon sterol-depletion. In contrast, in CHO-SCAP-eGFP-SPRING^{KO} cells, SCAP colocalizes with Giantin irrespective of the cellular sterol status. Representative images from three independent experiments are shown. (***B***). Cell viability of Hap1-WT and Hap1-SPRING^{KO} cells used for measuring cholesterol synthesis was determined using the MTT assay (N=8 biologically independent samples). Each bar represent the mean ± SEM.

Supplementary Figure 8

a

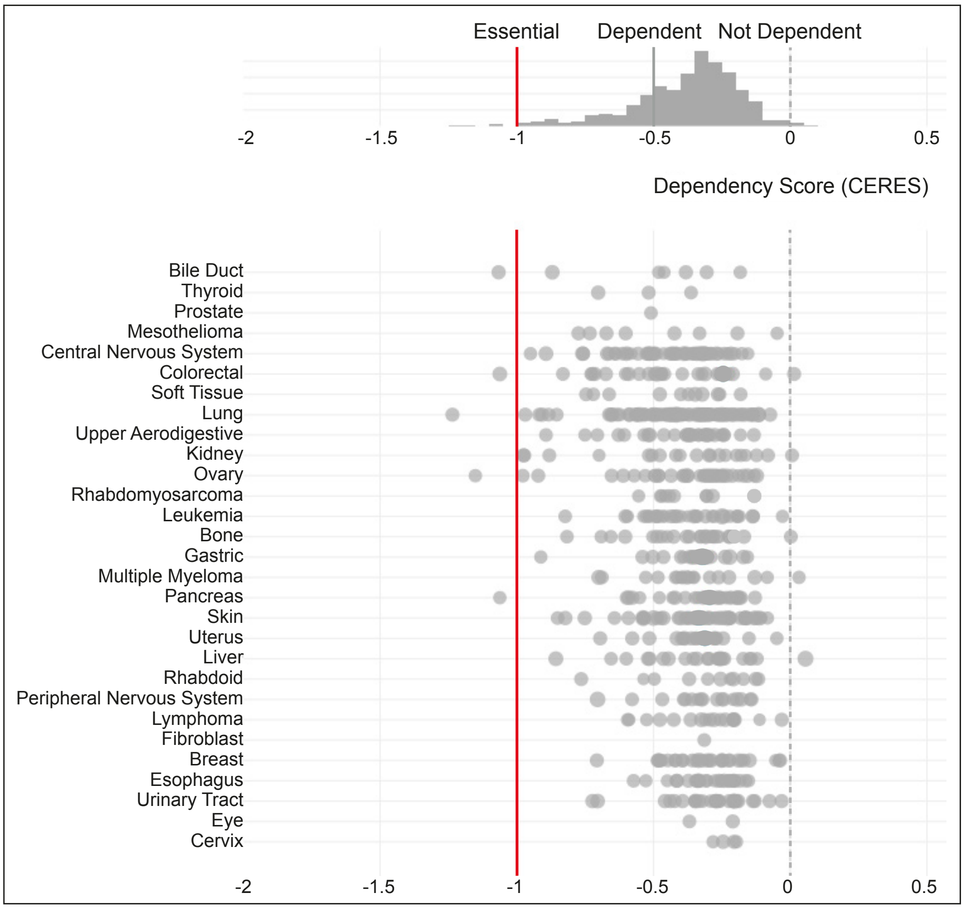

b

SPRING top Co-dependencies	
**Gene**	**Pearson correlation**
SREBF1	0.46
MBTPS1	0.43
SCAP	0.37
SCD	0.37
MBTPS2	0.36

Supplementary Figure 8. Analysis of the DepMap repository identifies dependency of cancer cell lines on *SPRING* expression. (*A*) Overview of the CERES score (*i.e.* dependency measure[1]) of cancer cell lines for *SPRING* expression within the CRISPR lethality project in the Avana public 19Q3 database (www.depmap.org). A CERES score below 0 increases the likelihood of a given cell line being dependent on *SPRING* expression. A score of 0 is considered to represent a gene that is not essential, whereas a score of -1 represents the median of known essential genes. Each point represents an individual cell line that belongs to the listed lineage. (***B***). The top 5 *SPRING* co-dependent genes in the aforementioned database are indicated with the corresponding Pearson correlation score.

1. Meyers, R. M. et al. Computational correction of copy number effect improves specificity of CRISPR-Cas9 essentiality screens in cancer cells. Nat. Genet. 49, 1779–1784 (2017).

Supplementary Figure 9

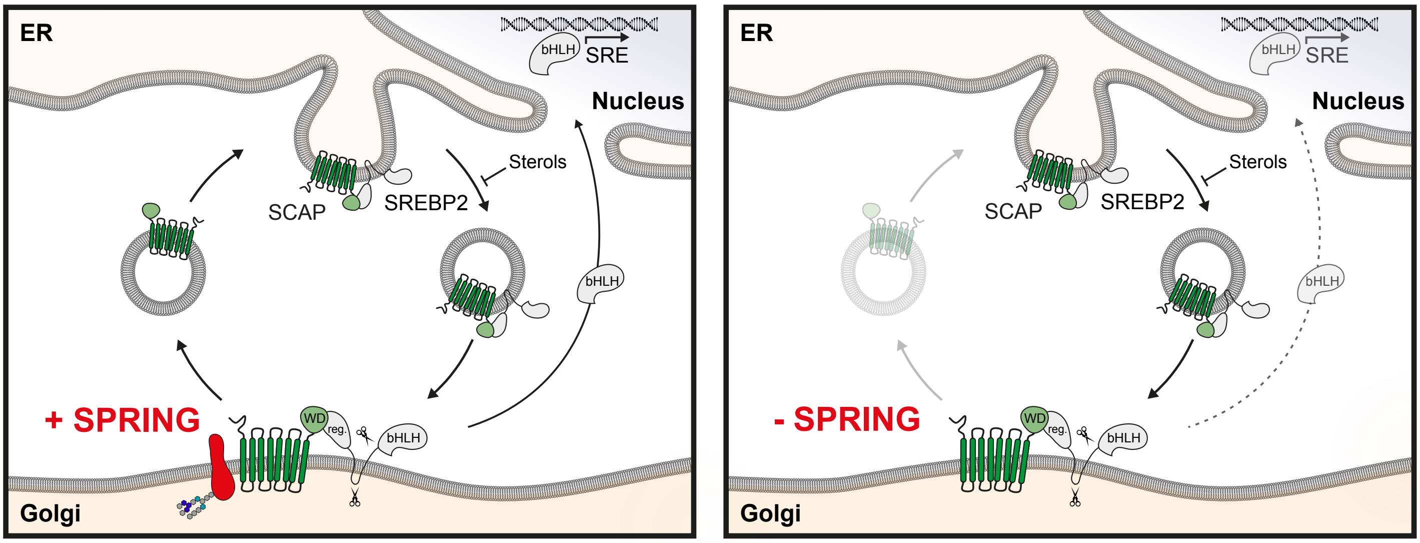

Supplementary Figure 9. Simplified schematic representation of regulation of SREBP signaling by *SPRING.* (*left*) In the presence of SPRING, SCAP can be retrieved to the ER. This ensures that SREBP signaling remains intact. (right) Absence of SPRING results in functional depletion of SCAP and attenuates SREBP signaling. Potential mechanisms underlying this may include regulation of S1P/S2P activity and/or control of SCAP recycling by SPRING, as elaborated on in the "Discussion" section.
