# Supplementary material for: Haploid genetic screens identify SPRING/C12ORF49 as a determinant of SREBP signaling and cholesterol metabolism
Source: Nat Commun. 2020 Feb 28;11:1128. doi: 10.1038/s41467-020-14811-1 (PMC7048761; doi:10.1038/s41467-020-14811-1)
Supplement: Supplementary file 8 — Reporting Summary [file 41467_2020_14811_MOESM8_ESM.pdf]

nature research

Corresponding author(s): Thijn Brummelkamp
Noam Zelcer

Last updated by author(s): 24/1/20

Reporting Summary

Nature Research wishes to improve the reproducibility of the work that we publish. This form provides structure for consistency and transparency in reporting. For further information on Nature Research policies, see Authors & Referees and the Editorial Policy Checklist.

Statistics

For all statistical analyses, confirm that the following items are present in the figure legend, table legend, main text, or Methods section.

n/a	Confirmed	
☐	☒	The exact sample size (*n*) for each experimental group/condition, given as a discrete number and unit of measurement
☐	☒	A statement on whether measurements were taken from distinct samples or whether the same sample was measured repeatedly
☐	☒	The statistical test(s) used AND whether they are one- or two-sided<br>*Only common tests should be described solely by name; describe more complex techniques in the Methods section.*
☒	☐	A description of all covariates tested
☐	☒	A description of any assumptions or corrections, such as tests of normality and adjustment for multiple comparisons
☐	☒	A full description of the statistical parameters including central tendency (e.g. means) or other basic estimates (e.g. regression coefficient) AND variation (e.g. standard deviation) or associated estimates of uncertainty (e.g. confidence intervals)
☐	☒	For null hypothesis testing, the test statistic (e.g. *F*, *t*, *r*) with confidence intervals, effect sizes, degrees of freedom and *P* value noted<br>*Give P values as exact values whenever suitable.*
☒	☐	For Bayesian analysis, information on the choice of priors and Markov chain Monte Carlo settings
☒	☐	For hierarchical and complex designs, identification of the appropriate level for tests and full reporting of outcomes
☒	☐	Estimates of effect sizes (e.g. Cohen's *d*, Pearson's *r*), indicating how they were calculated

Our web collection on statistics for biologists contains articles on many of the points above.

Software and code

Policy information about availability of computer code

Data collection	ImageQuant LAS4000 mini V1.3 (Fuji), CytExpert1.2 (Beckman Coulter), LightCycler 480 1.5.1.62 SP3 (Roche) , Xcalibur 4.1 (Thermo scientific), LCS Leica Application Suite X 3.7.1.21655 (Leica)
Data analysis	Prism Graphpad V6

For manuscripts utilizing custom algorithms or software that are central to the research but not yet described in published literature, software must be made available to editors/reviewers. We strongly encourage code deposition in a community repository (e.g. GitHub). See the Nature Research guidelines for submitting code & software for further information.

Data

Policy information about availability of data

All manuscripts must include a data availability statement. This statement should provide the following information, where applicable:

- Accession codes, unique identifiers, or web links for publicly available datasets
- A list of figures that have associated raw data
- A description of any restrictions on data availability

A full data availability section is provided in the manuscript. The datasets associated with this study are indicated in this section and have been made publicly available.

Field-specific reporting

Please select the one below that is the best fit for your research. If you are not sure, read the appropriate sections before making your selection.

☒ Life sciences ☐ Behavioural & social sciences ☐ Ecological, evolutionary & environmental sciences

For a reference copy of the document with all sections, see nature.com/documents/nr-reporting-summary-flat.pdf

Life sciences study design

All studies must disclose on these points even when the disclosure is negative.

Sample size	Sample size in in vitro experiments are detailed in the manuscript. For the animal experiments, we based the group sample size on a power calculations using parameters established in previous studies (e.g PMID: 19520913). This is also in line with the typical group size (5-8 mice) that is used by others in the field and is appropriate for the experimental setup.
Data exclusions	2 mice were excluded as the administered treatment (viral injection) failed (no transgene achieved). We test for outliers in experiments. One mouse was excluded as the majority of the measured values were identified as outliers using criteria established during analysis (PRISM V6, Grubbs, alfa=0.1).
Replication	Experiments were repeated as indicated above as detailed in the manuscript. Moreover, a large set of experiments were independently performed and successfully replicated in two different institutes by different investigators (NKI & AMC).
Randomization	Randomization was only relevant for the animal experiment (fig 4), where WT mice were infected with 2 distinct adenoviral constructs. In this experiment mice were randomly assigned to one of the two experimental groups.
Blinding	No blinding was applied in the experiments.

Reporting for specific materials, systems and methods

We require information from authors about some types of materials, experimental systems and methods used in many studies. Here, indicate whether each material, system or method listed is relevant to your study. If you are not sure if a list item applies to your research, read the appropriate section before selecting a response.

Materials & experimental systems

n/a	Involved in the study	
☐	☒	Antibodies
☐	☒	Eukaryotic cell lines
☒	☐	Palaeontology
☐	☒	Animals and other organisms
☒	☐	Human research participants
☒	☐	Clinical data

Methods

n/a	Involved in the study	
☒	☐	ChIP-seq
☐	☒	Flow cytometry
☒	☐	MRI-based neuroimaging

Antibodies

Antibodies used

the antibodies used in the study are listed in supplementary table 4 (key resources) and are listed here below again.
SPRING (C12orf49) Atlas Antibodies #HPA026905
LDLR Biovision #3839
SQLE Proteintech #12544-1-AP
HMGCR ATCC CRL-1811 IgG-A9 (undiluted hybridoma supernatant)
SREBP2 N-term clone 22D5, millipore #MABS1988
SREBP2 C-term BD Bioscience #557037
ß-actin Merck #MAB1501
CDK4 Santa Cruz #sc-260
Flag-M2 Sigma Aldrich #F1804
FASN Santa Cruz #sc-55580
Histone H3 Abcam #ab1791
SREBP1 N-term clone 20B12, millipore #MABS1987
GFP B-2, Santa Cruz #sc-9996
GFP (for IPs) Roche #11814460001
Transferrin receptor clone H68.4, Invitrogen #13-6800
VAPA kind gift from Sjaak Neefjes -
GM130 Cell Signaling #12480
LDLR-APC R&D #472413
SCAP Santa Cruz #sc-9675

SCAP Bethyl Laboratories #A303-554A
S1P Arigo ARG56137
C12ORF49/SPRING Thermo Fisher #PA5-55459

Validation	Used antibodies are listed above. These antibodies were validated by the commercial providers and additionally in our previous studies (PMID: 31117816, 31327168, 30601691, 30658189, 29903737, 28882874, 28231341 etc). Specifically, in Supplementary figure II-D we validate one of the antibodies used in the current study to demonstrate its specificity (SCAP, Bethyl Laboratories, #A303-554A).

Eukaryotic cell lines

Policy information about cell lines

Cell line source(s)	As indicated in the M&M section, the cells used in the study were obtained from the ATCC (HeLa, HEK293T, Hepa1-6). The CHO-SCAP-eGFP cells were from Dr. P. Espenshade. The Hap1 cells used for the genetic screens were from the participating NKI group.
Authentication	With the exception of the Hap1 cells, which are uniquely haploid as repeatedly confirmed by evaluating DNA content with propidium iodide staining, all other cells were not confirmed.
Mycoplasma contamination	All cells were regularly tested for mycoplasma infection and were negative
Commonly misidentified lines (See ICLAC register)	No commonly misidentified lines were used.

Animals and other organisms

Policy information about studies involving animals; ARRIVE guidelines recommended for reporting animal research

Laboratory animals	In the study, male, ±8 wk old C57BL/6J mice (Charles River) and Rosa26-LSL-Cas9 knock-in mice (#02857, The Jackson Laboratory) were used. Mice were fed a standard chow diet and housed in a temperature-controlled room under a 12-hour light-dark cycle under pathogen-free conditions
Wild animals	No wild animals were used in the study.
Field-collected samples	No field-collected animals were used in the study.
Ethics oversight	Ethical approval for the animal experiments falls under experimental protocols approved by the Dutch National Committee for Animal Experiments to PI NZ, and supervision by the local ethical committee of the NKI and AMC.

Note that full information on the approval of the study protocol must also be provided in the manuscript.

Flow Cytometry

Plots

Confirm that:

☒ The axis labels state the marker and fluorochrome used (e.g. CD4-FITC).

☒ The axis scales are clearly visible. Include numbers along axes only for bottom left plot of group (a 'group' is an analysis of identical markers).

☒ All plots are contour plots with outliers or pseudocolor plots.

☒ A numerical value for number of cells or percentage (with statistics) is provided.

Methodology

Sample preparation	The procedure is detailed in the M&M section; Cells were detached, washed with FACS buffer (2 mM EDTA, 0.5 % BSA in PBS) and then then fixed with 4% paraformaldehyde before measurement. For measuring surface LDLR cells were stained with anti-LDLR-APC before the fixation step.
Instrument	CytoFlex (Beckman Coulter)
Software	CytExpert1.2 (Beckman Coulter)
Cell population abundance	In the study only samples form the Hap1 cell line were subjected to FACS analysis. This cell population was uniform and represented all the analyzed cells.
Gating strategy	The only gating used for analyzing Hap1 cells by FACS (above) is a gate to exclude cell debris. All other cells were included in the analysis.

☒ Tick this box to confirm that a figure exemplifying the gating strategy is provided in the Supplementary Information.
