# Supplementary material for: Haploid genetic screens identify SPRING/C12ORF49 as a determinant of SREBP signaling and cholesterol metabolism
Source: Nat Commun. 2020 Feb 28;11:1128. doi: 10.1038/s41467-020-14811-1 (PMC7048761; doi:10.1038/s41467-020-14811-1)

Figure 1B

SQLE

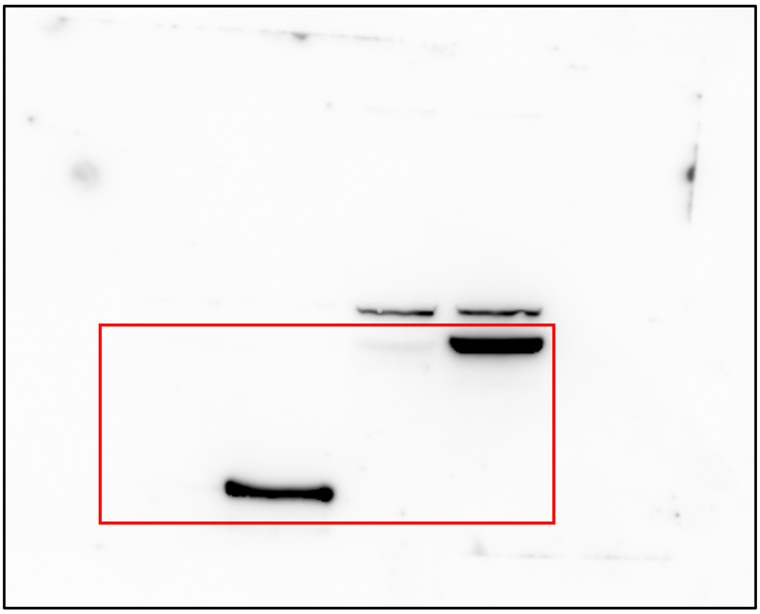

Actin

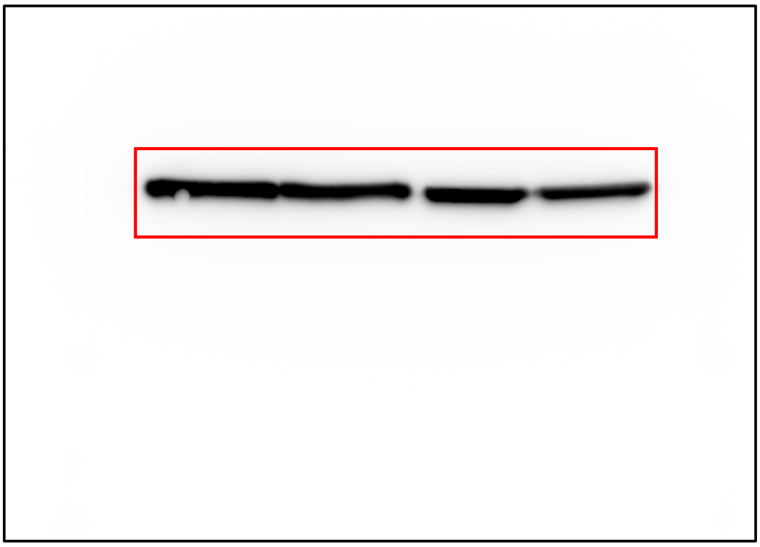

Figure 2B

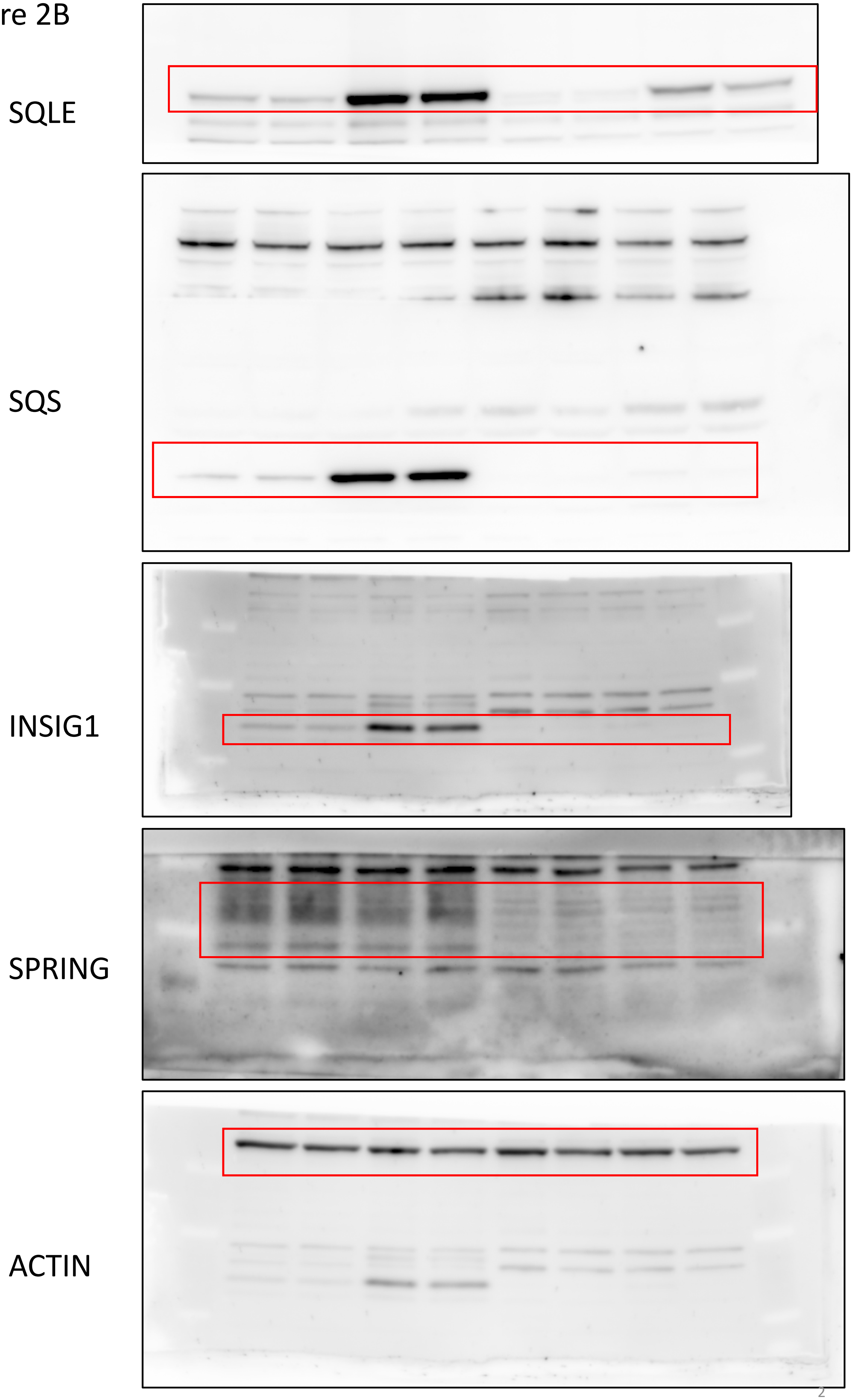

Figure 2E

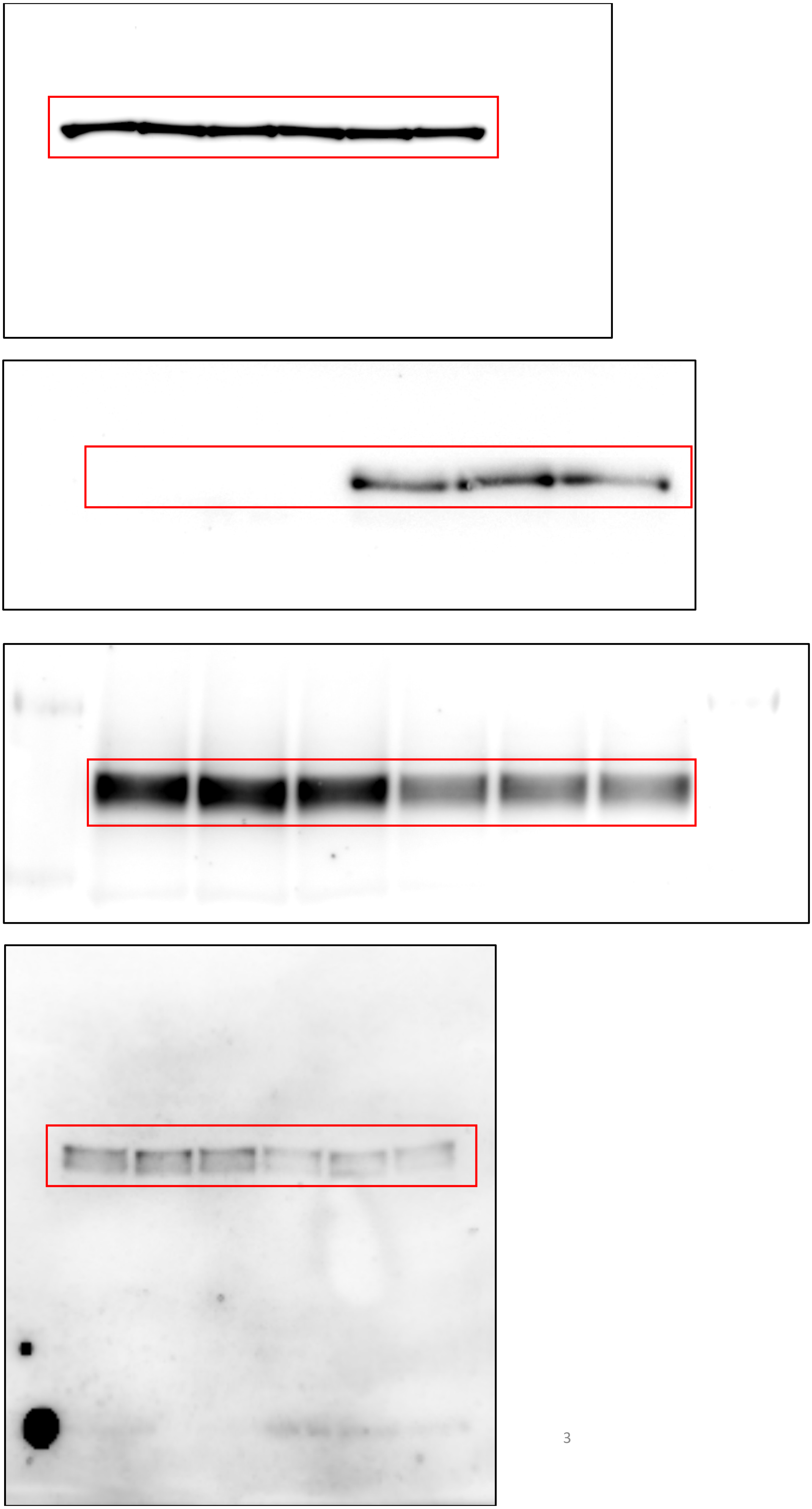

Actin

GFP

HMGCR

SREBP1 N

Figure 2E

SREBP2 N

TfR

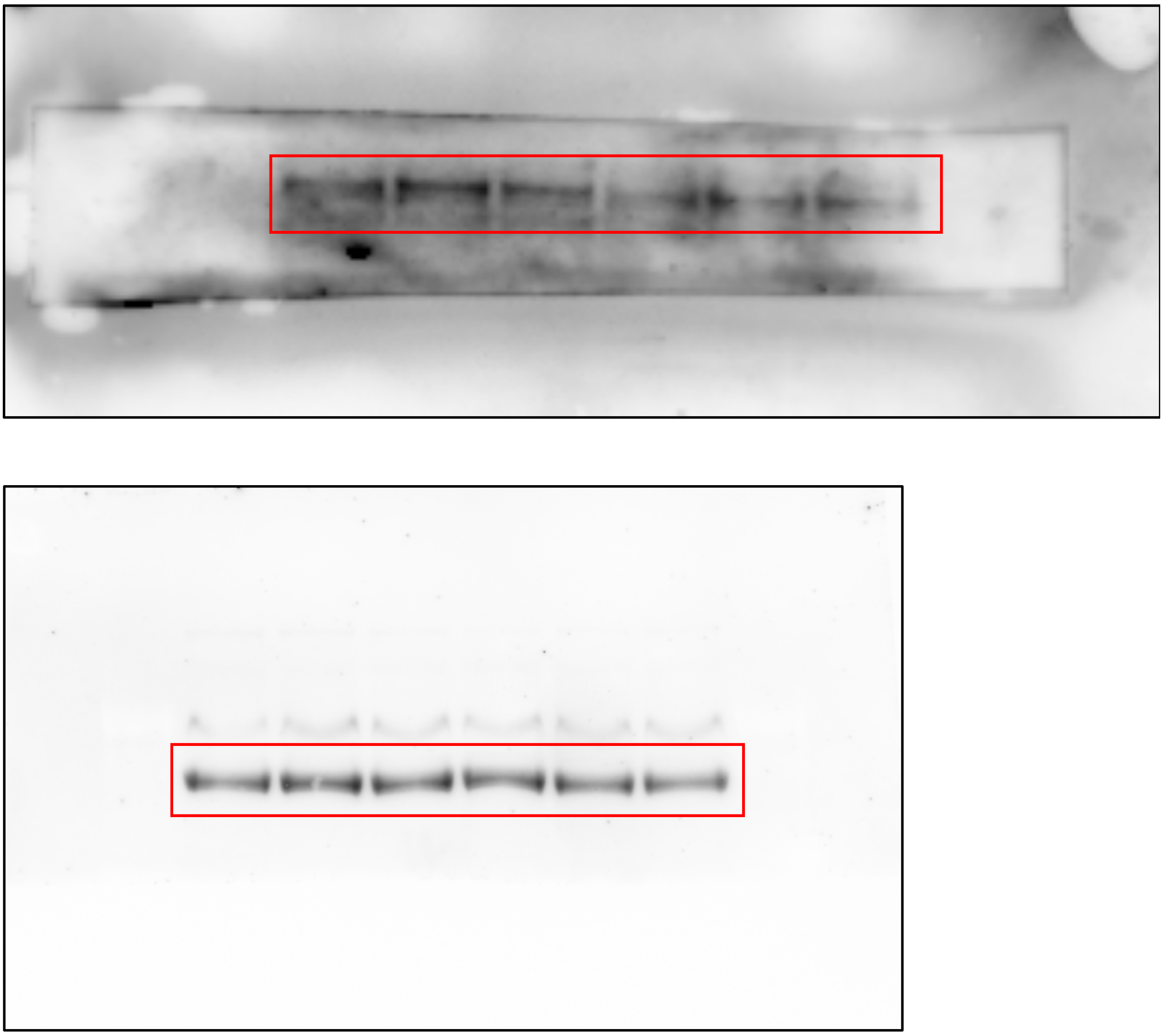

Figure 5C

SPRING

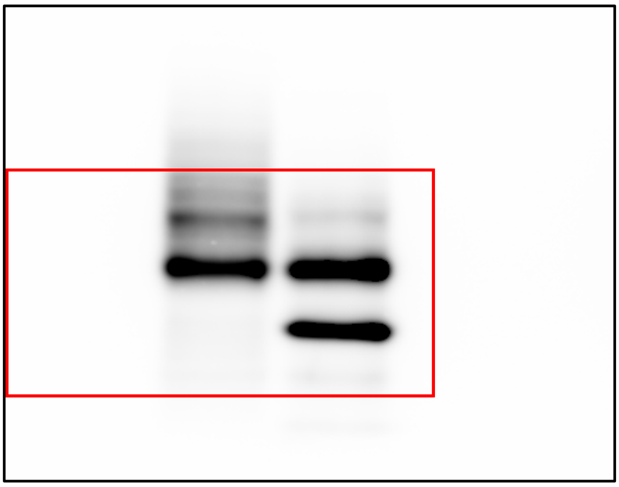

TUBULIN

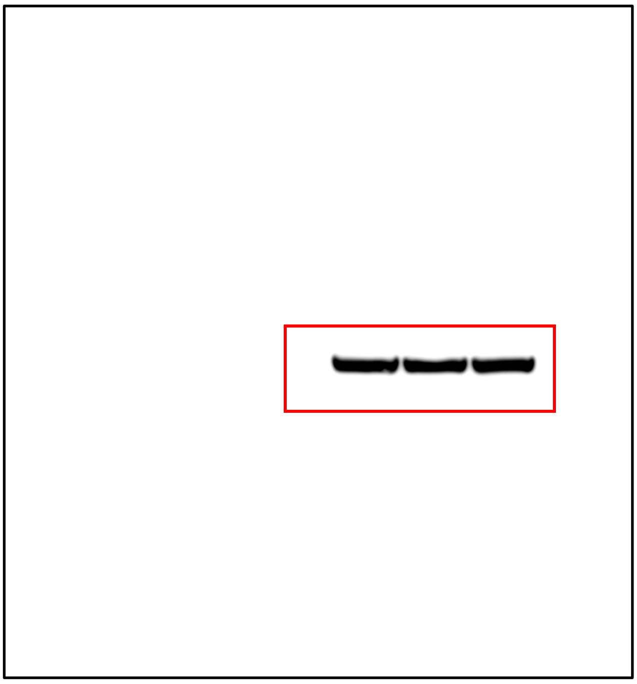

Figure 5D

HA

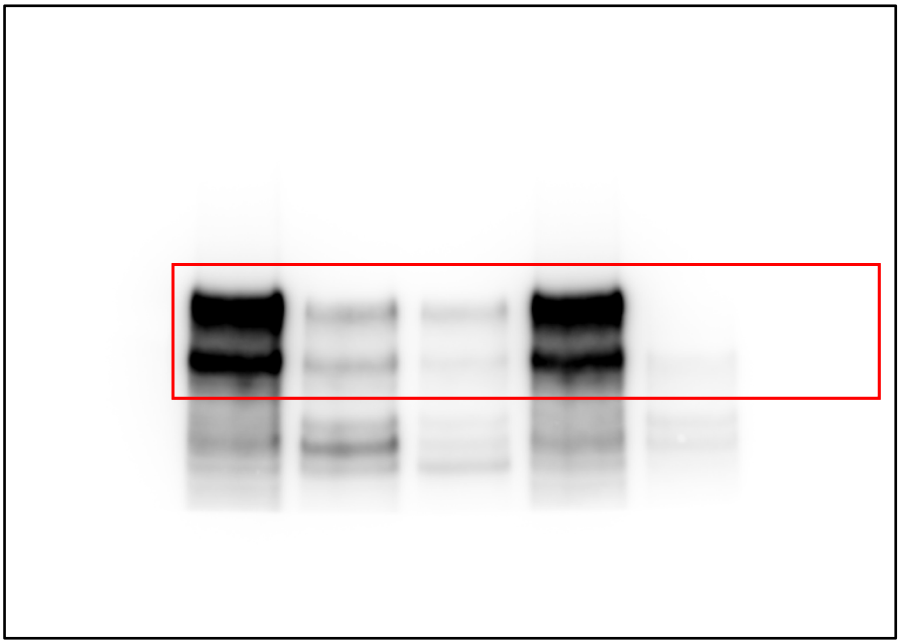

V5

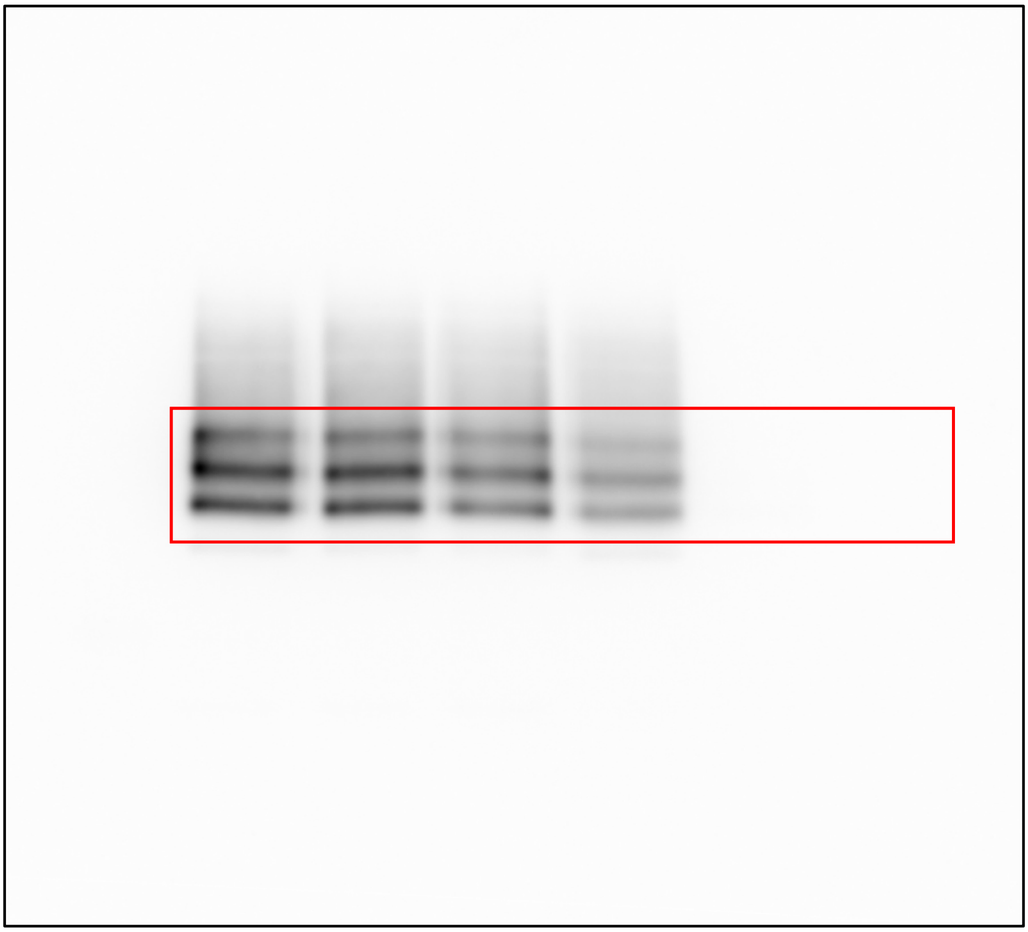

Figure 6A

GFP

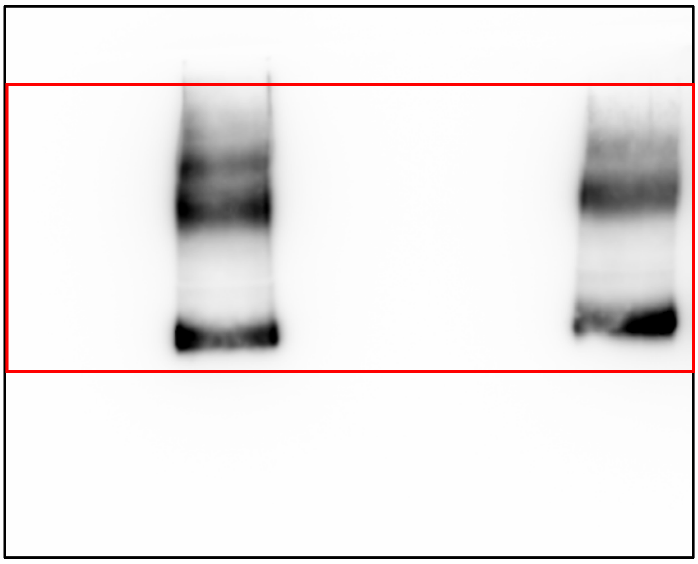

V5

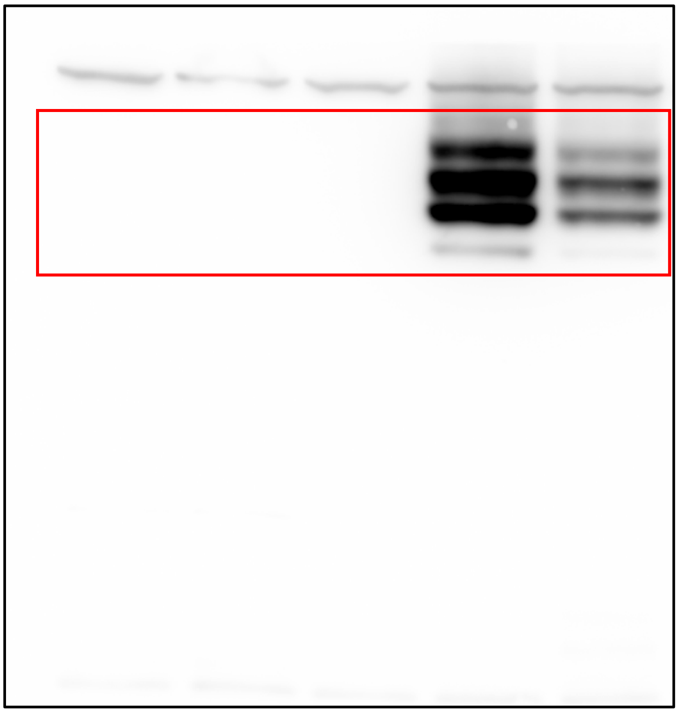

V5 - IP

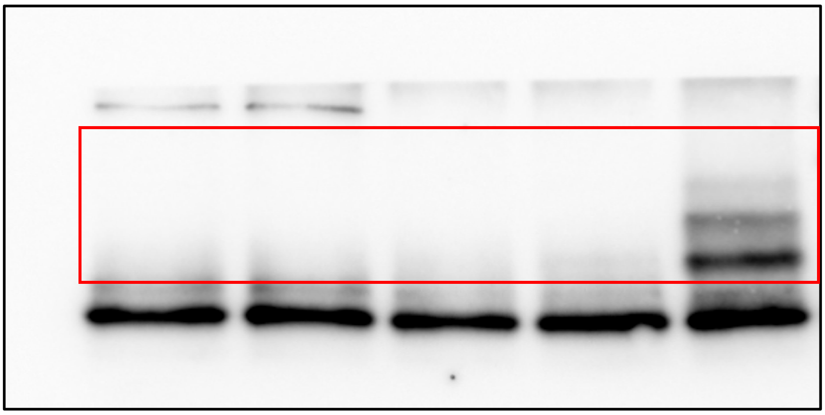

Figure 6A

IgG

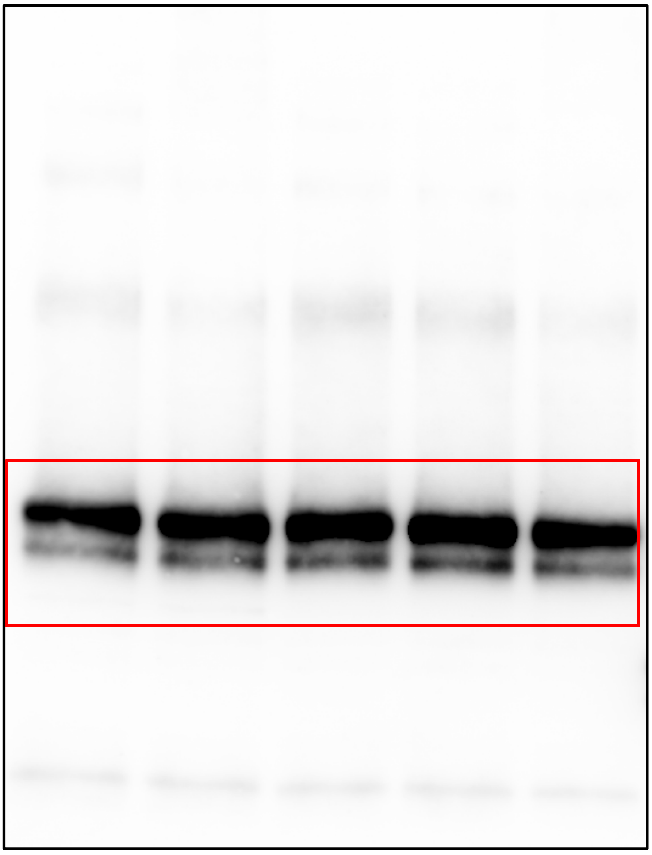

GFP (IP)

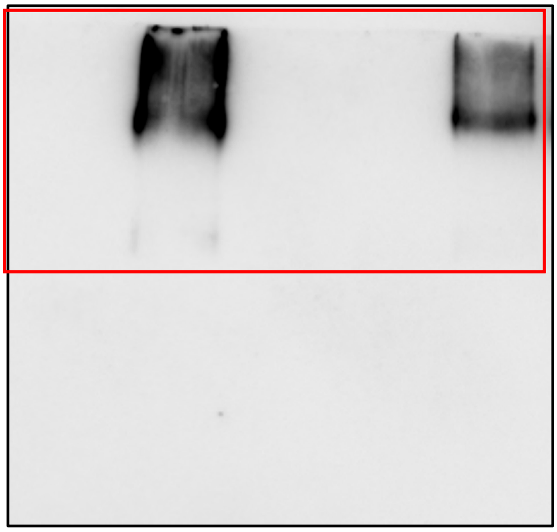

Figure 6C

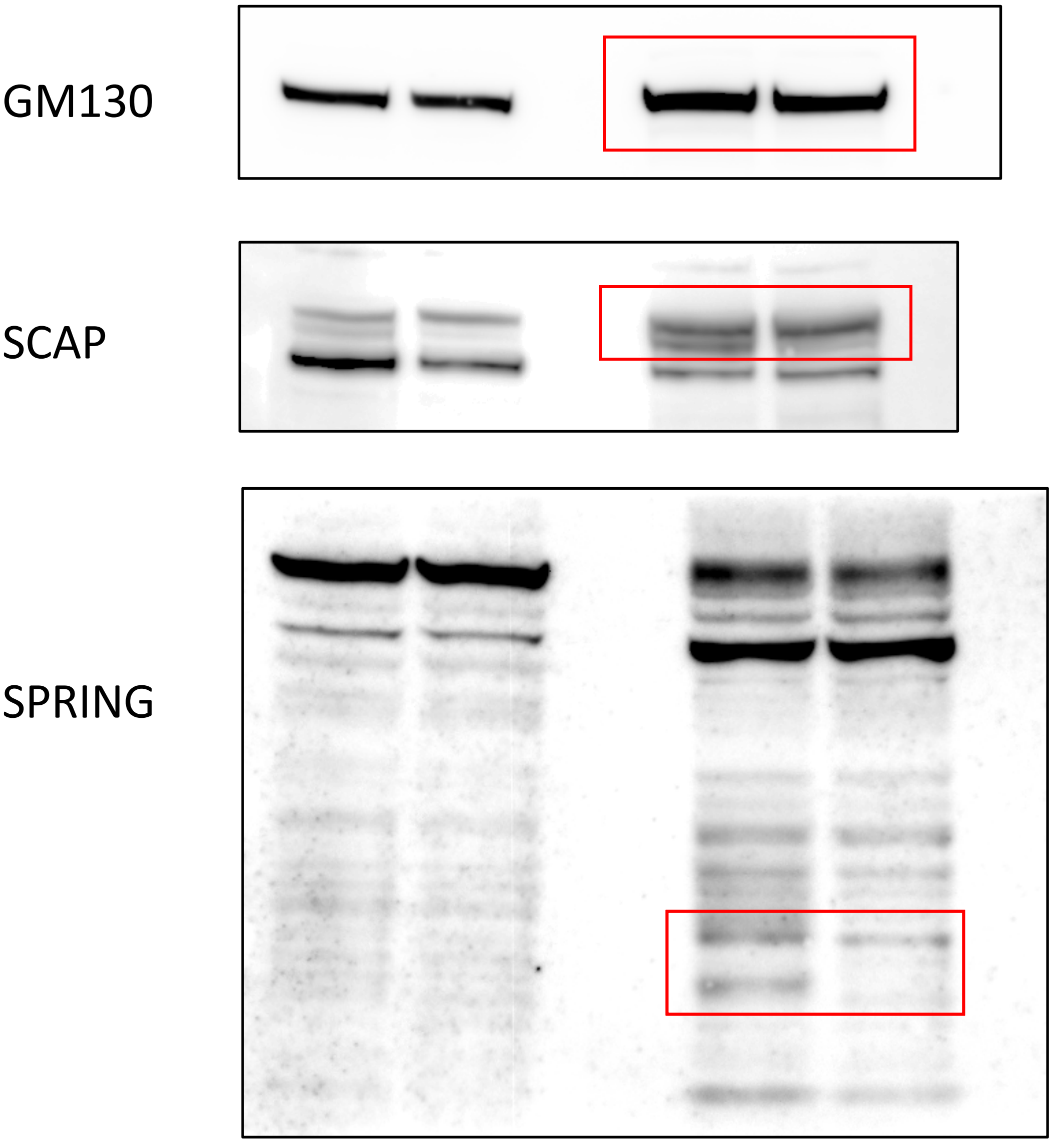

Figure 6D

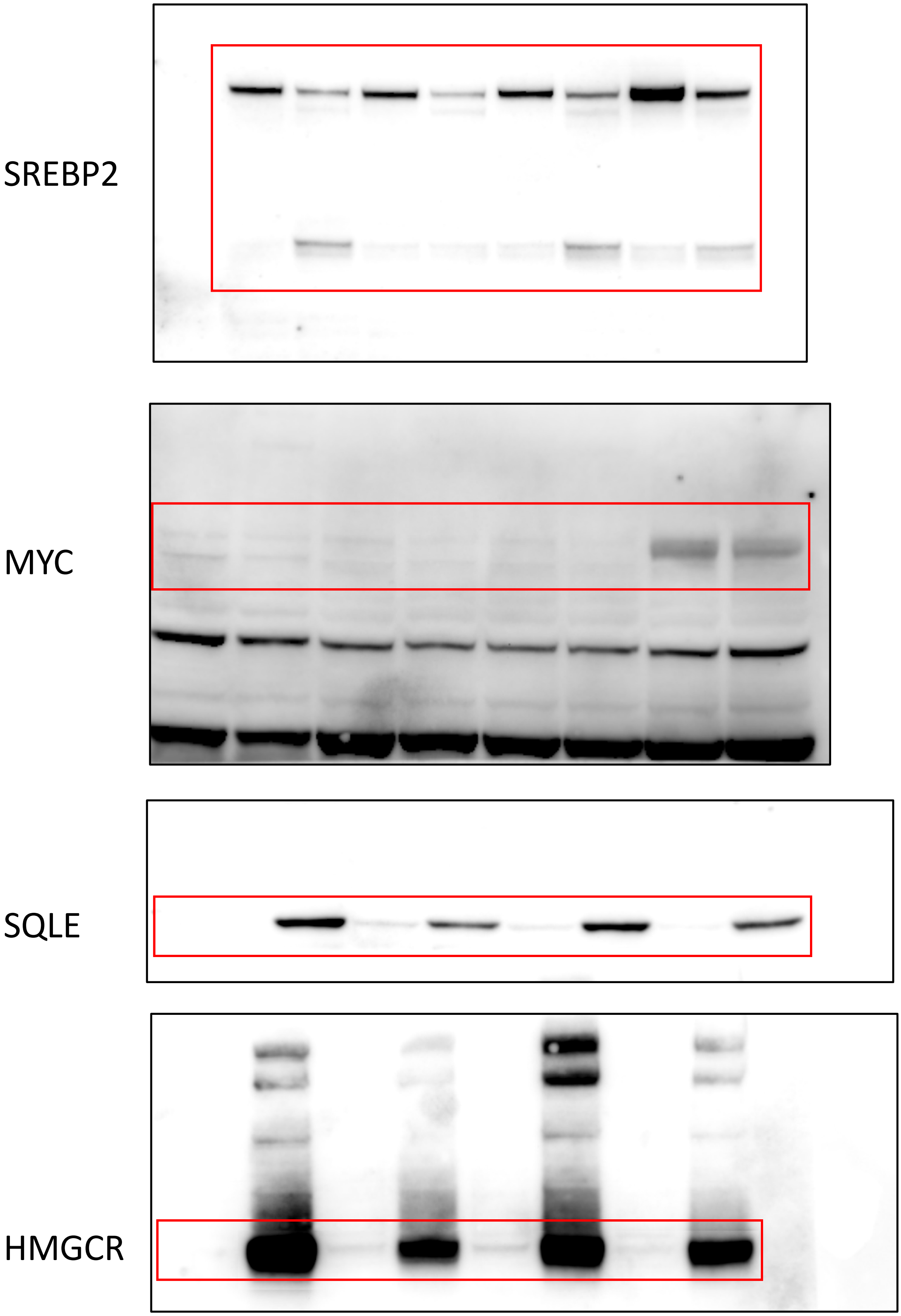

Figure 6D

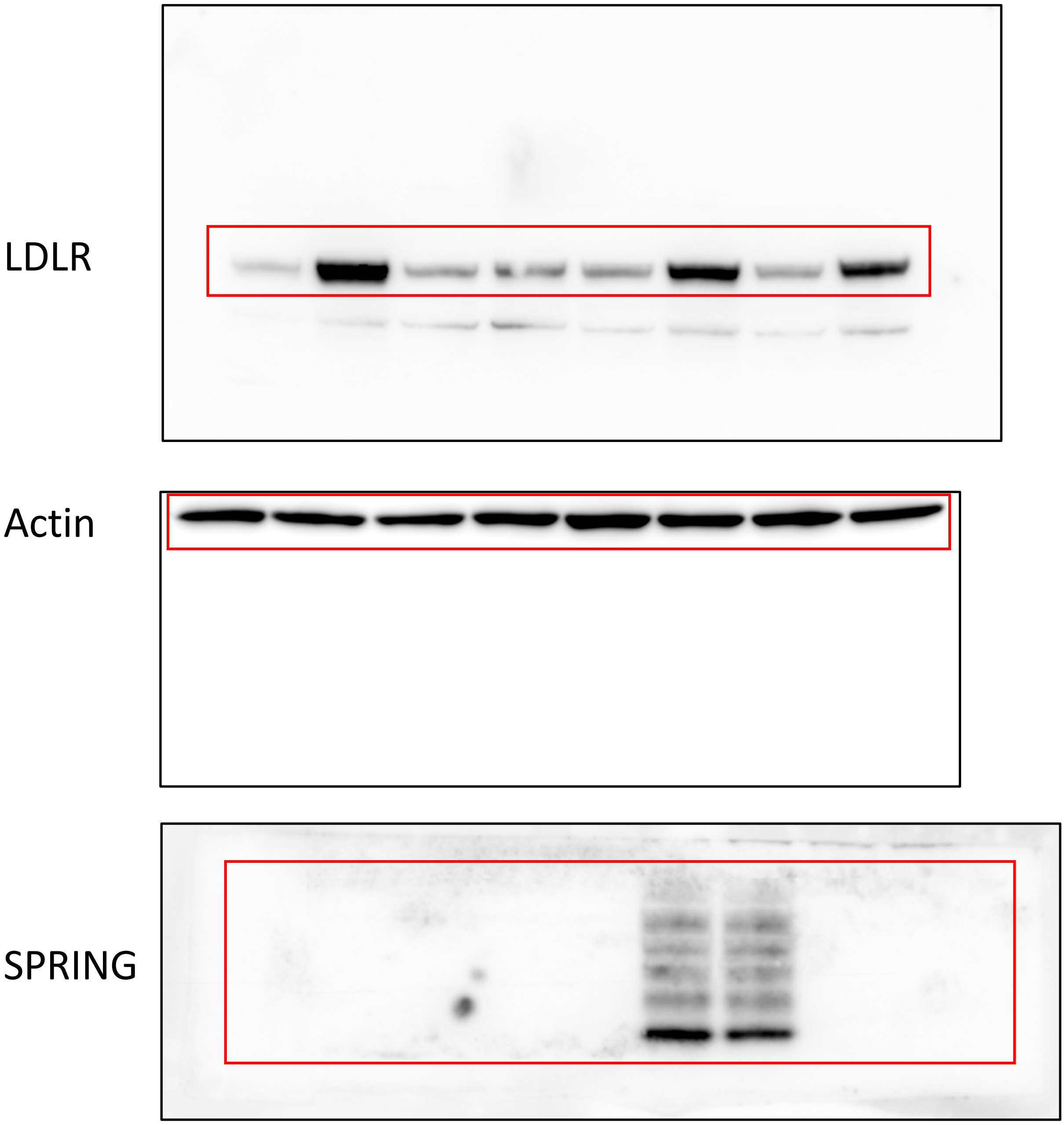

Supplementary Figure 1B

SQLE

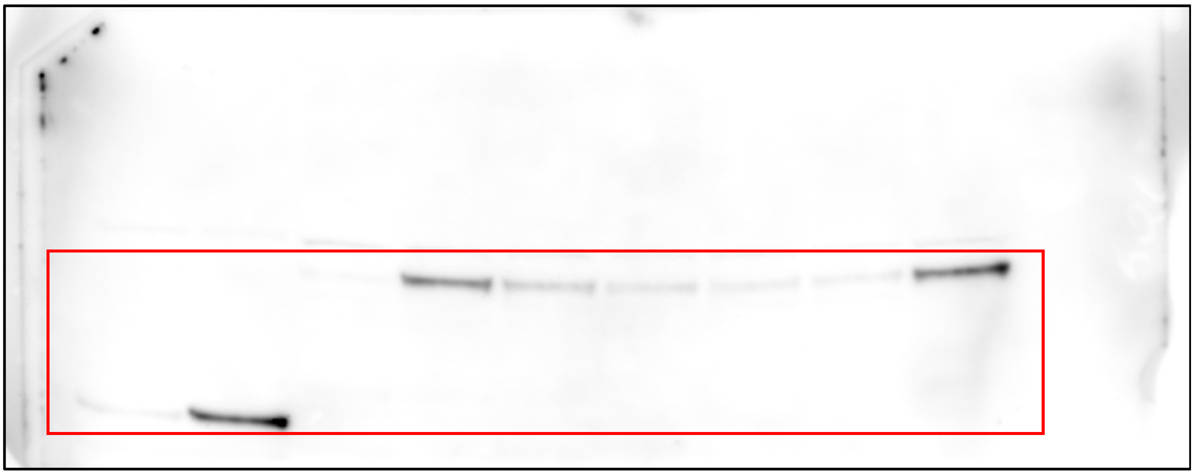

Actin

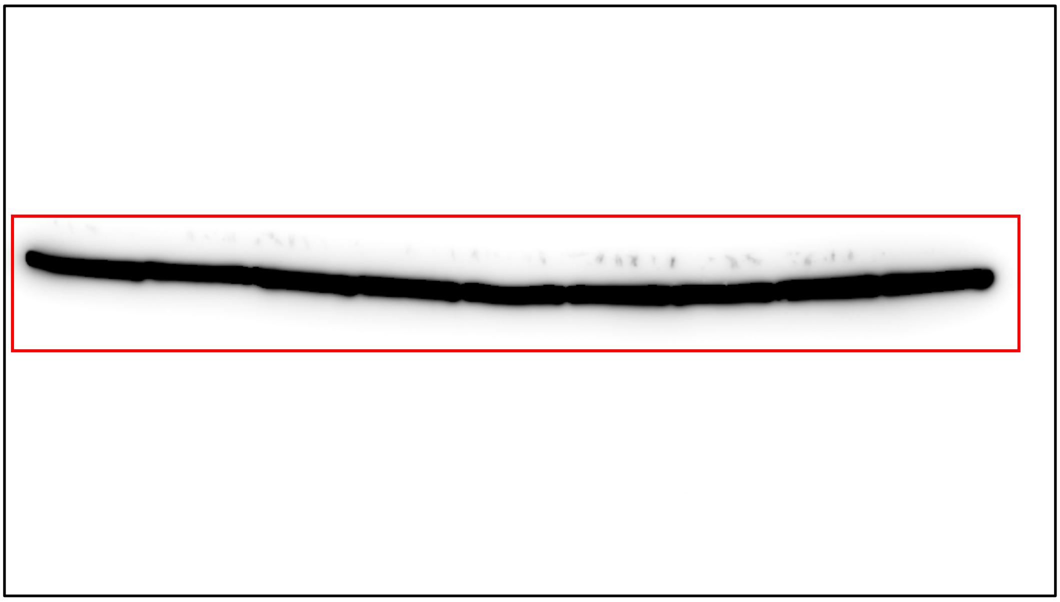

Supplementary Figure 2B

S1P

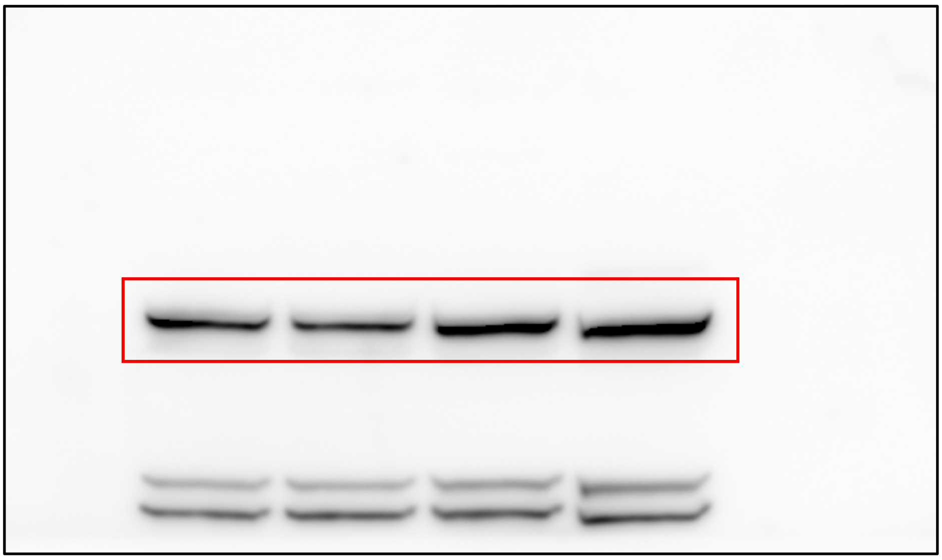

ACTIN

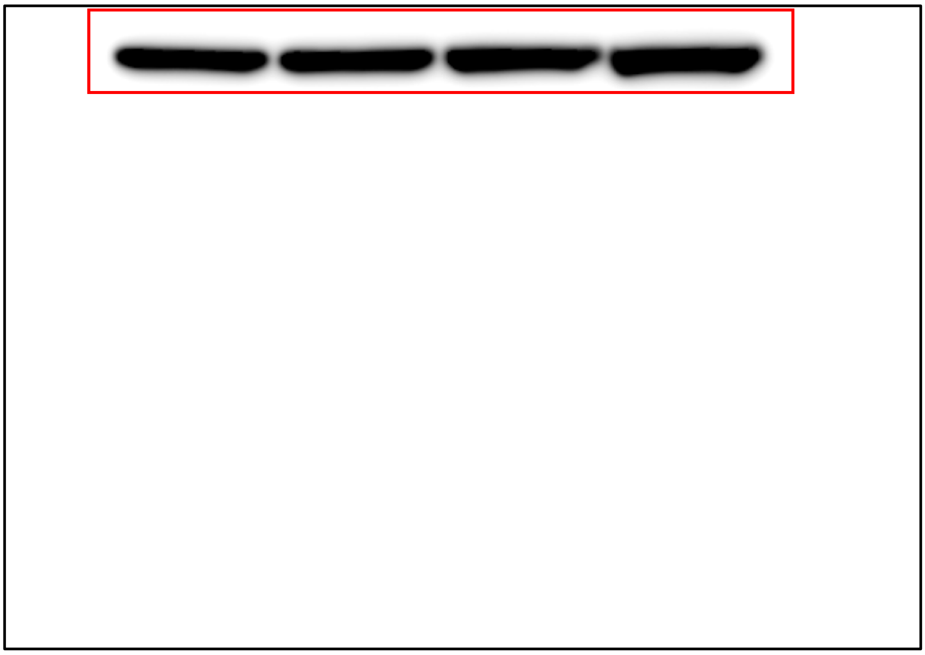

Supplementary Figure 2D

SCAP

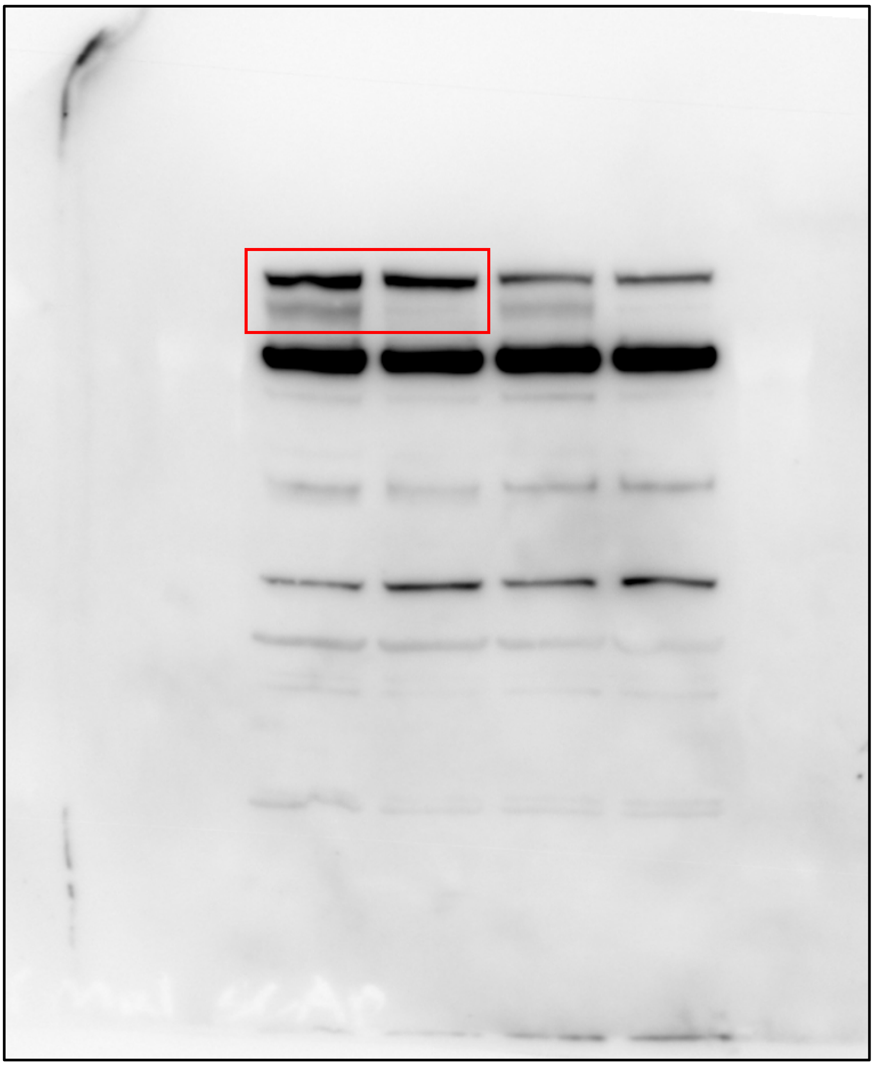

ACTIN

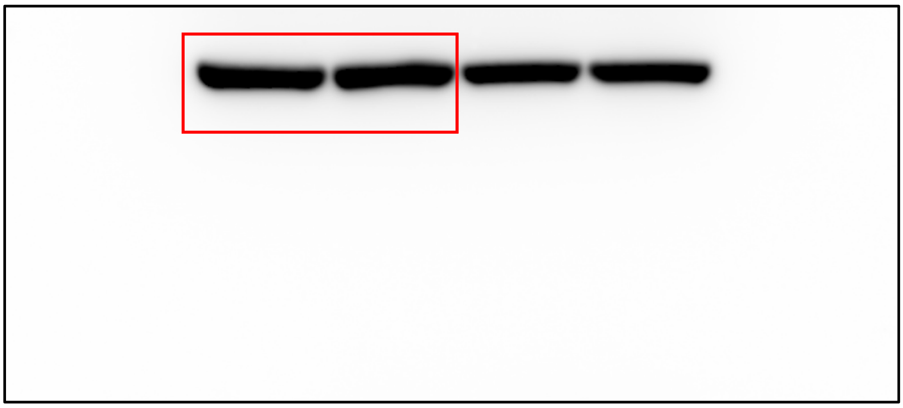

Supplementary Figure 4B

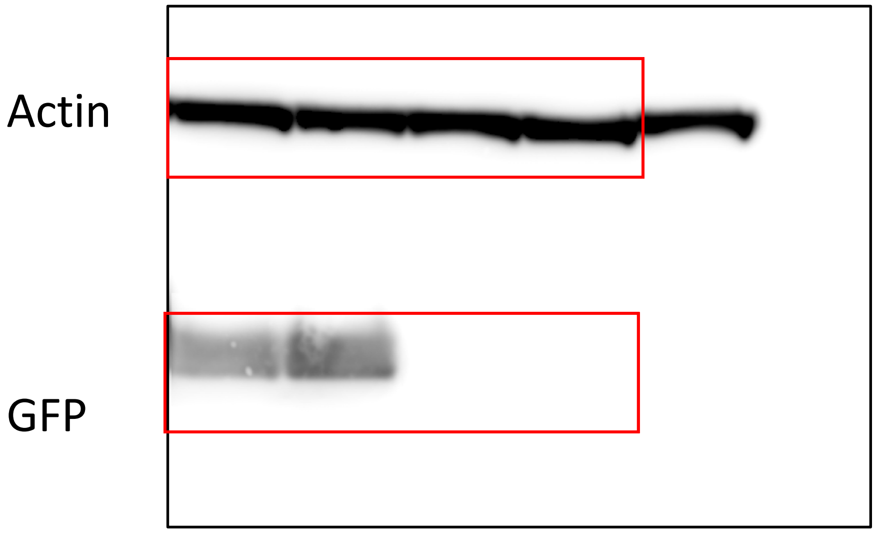

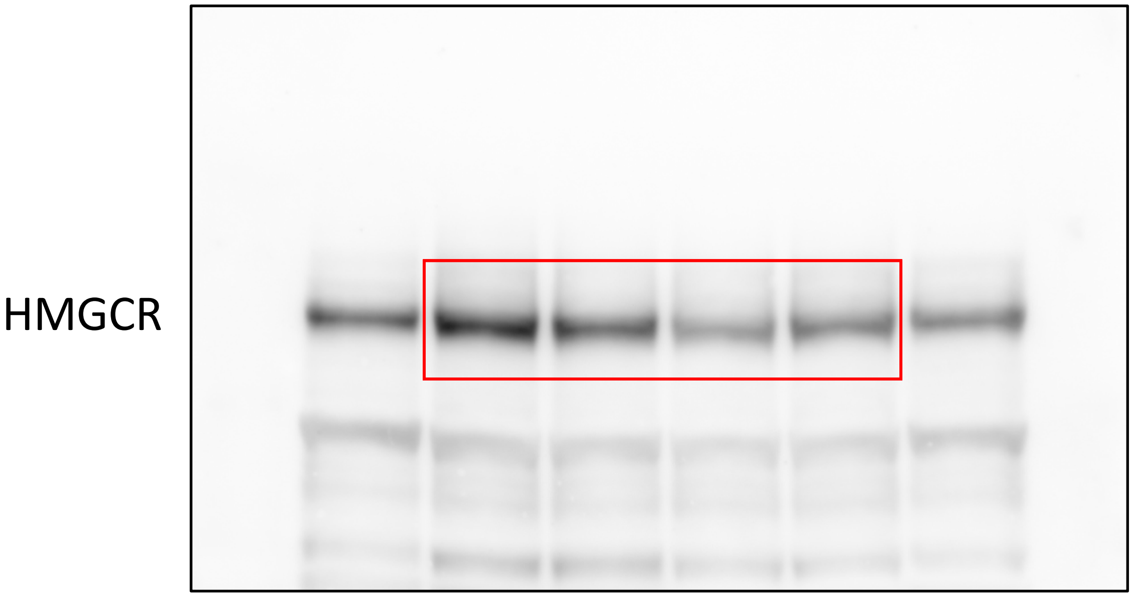

Supplementary Figure 4B

LDLR

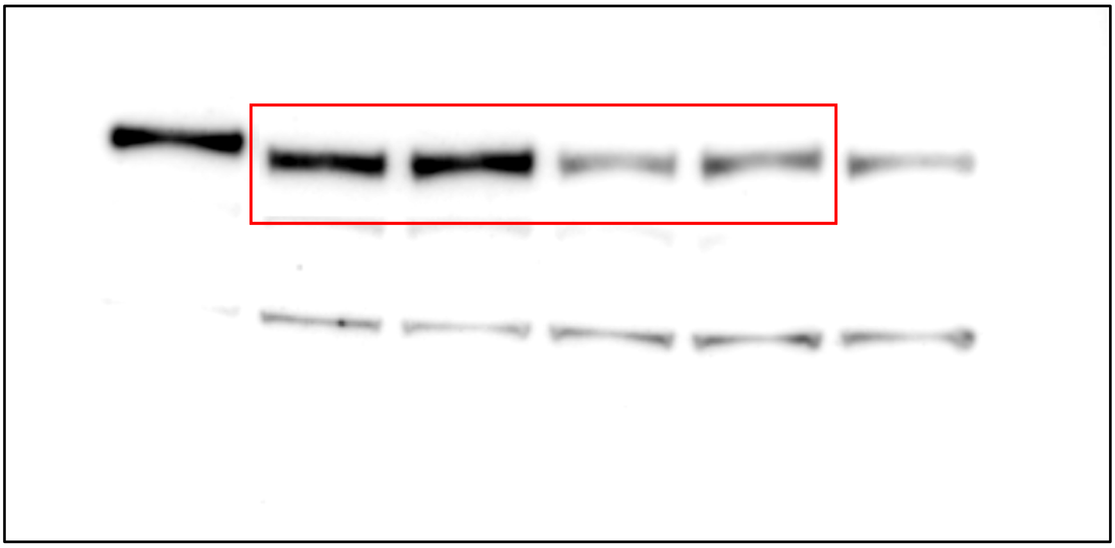

SREBP1

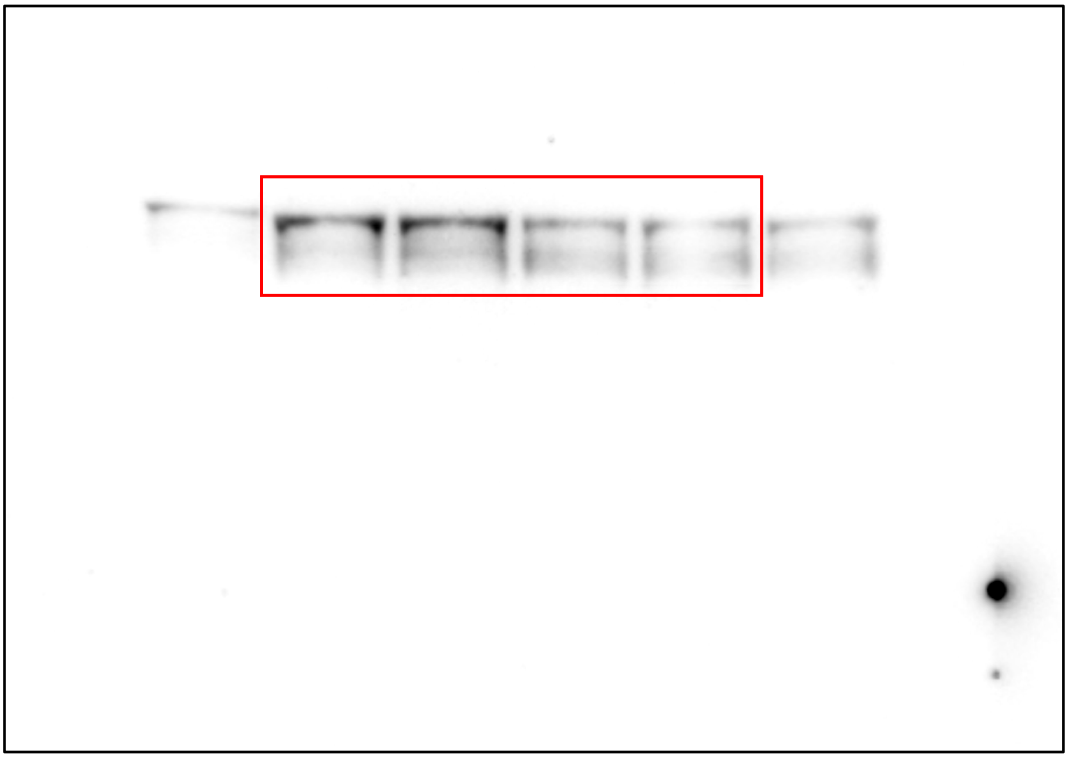

Supplementary Figure 4B

SREBP2

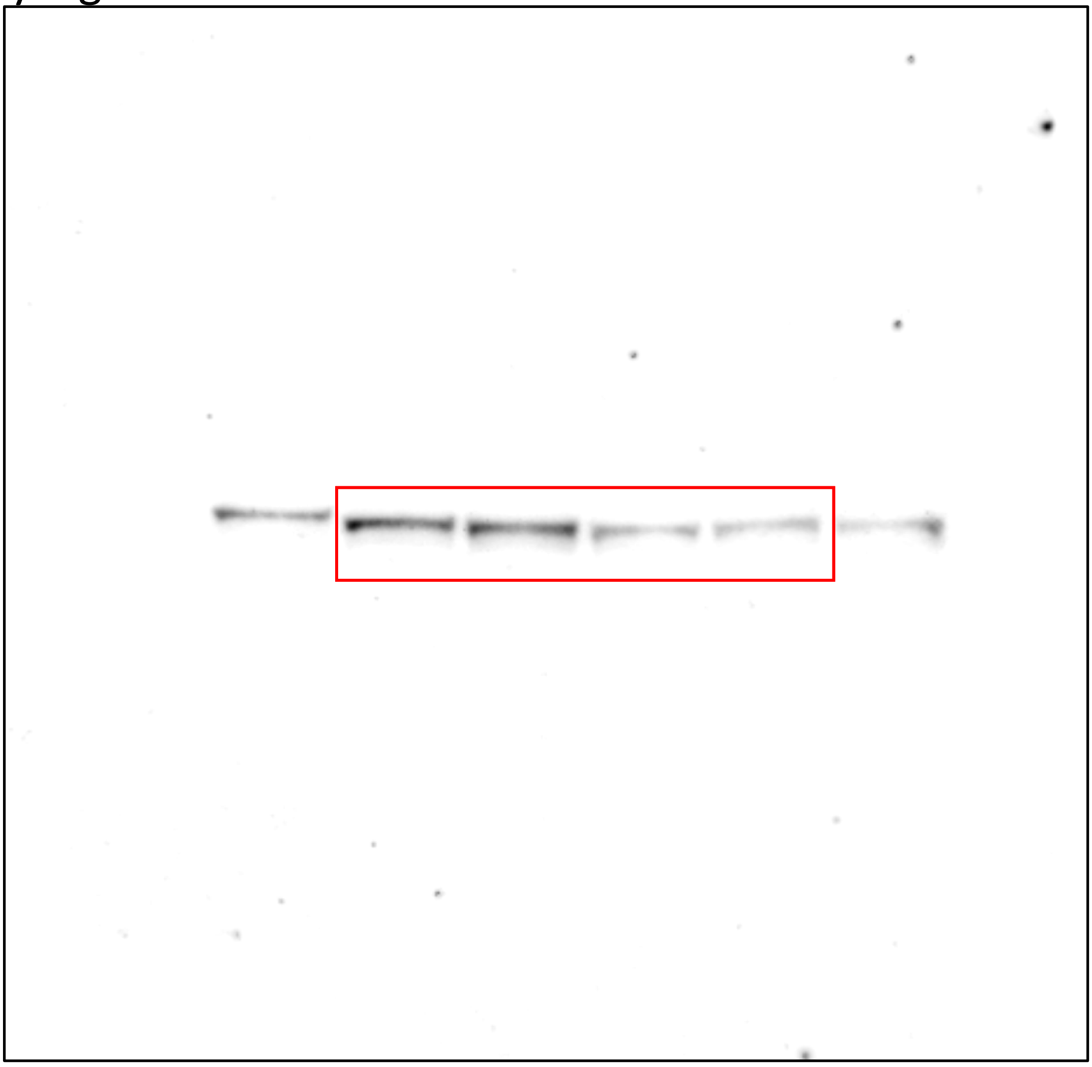

TfR

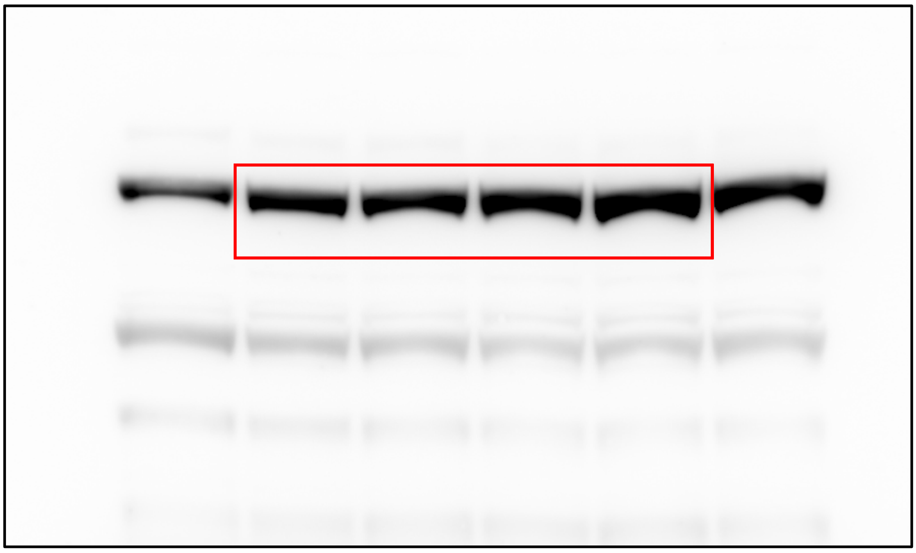

Supplementary Figure 4C

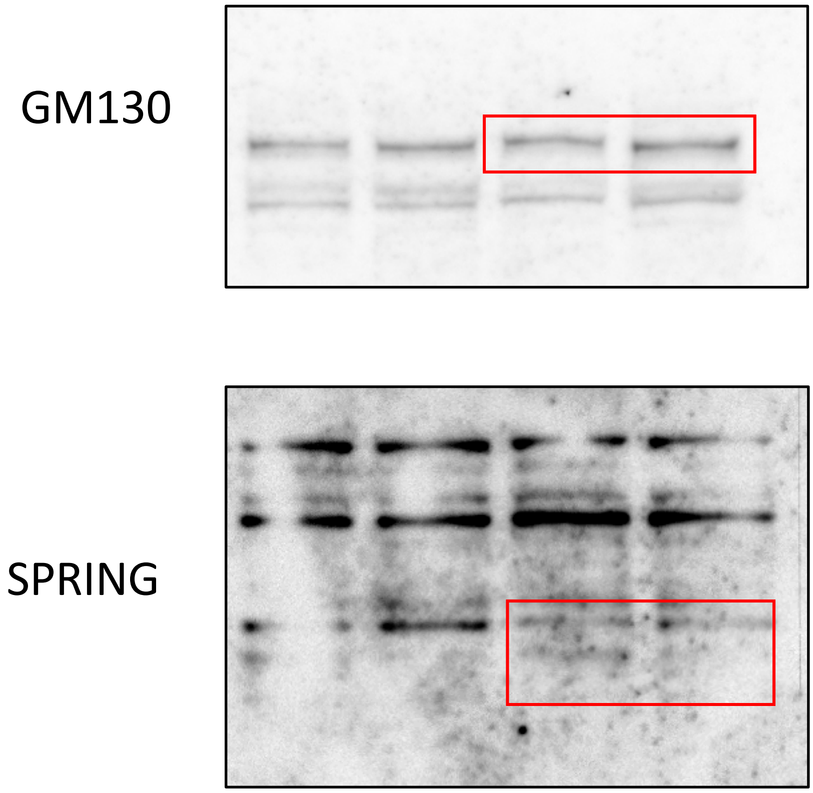

Supplementary Figure 6A

GM130

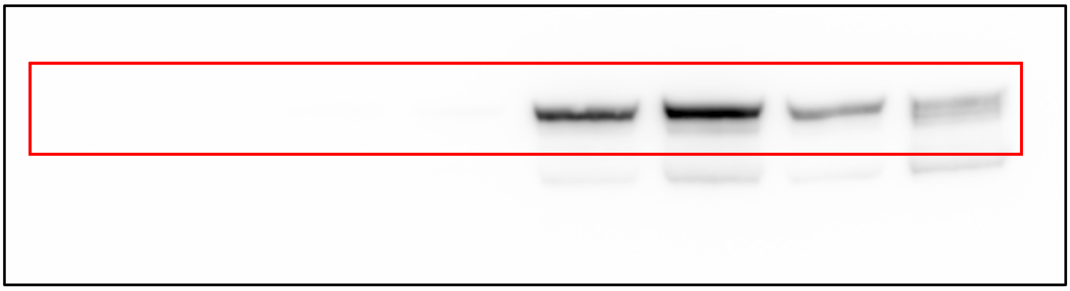

SPRING

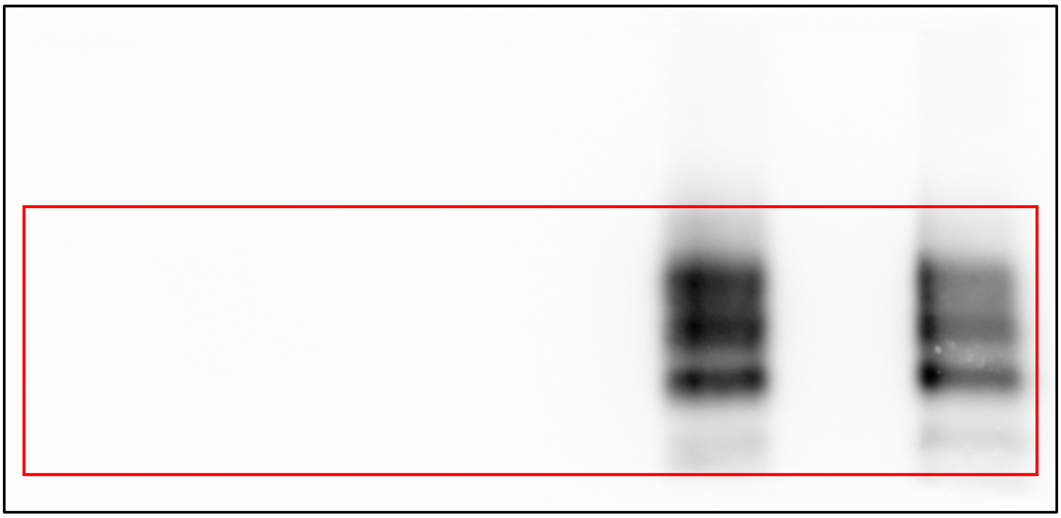

TUBULIN

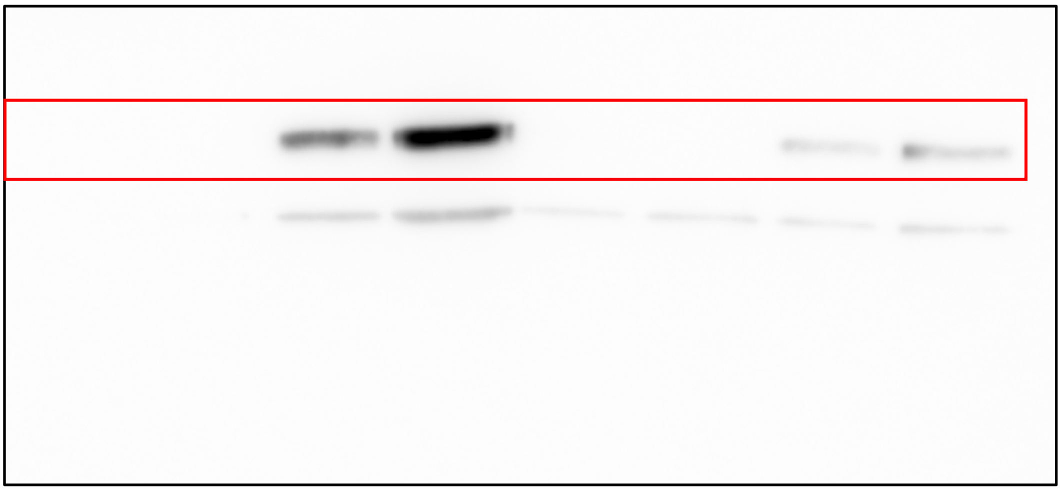

Supplementary Figure 6B

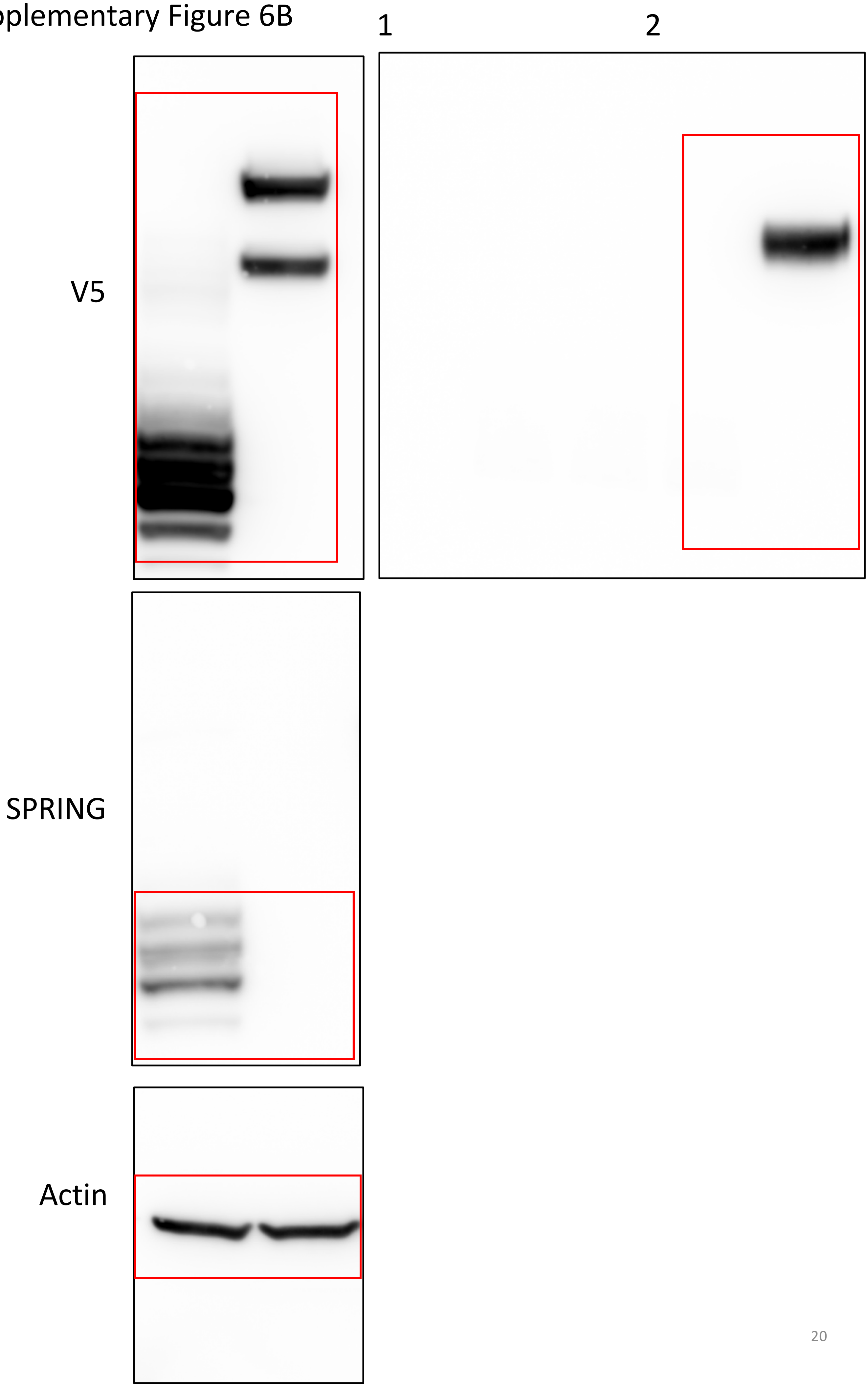

Supplementary Figure 6C

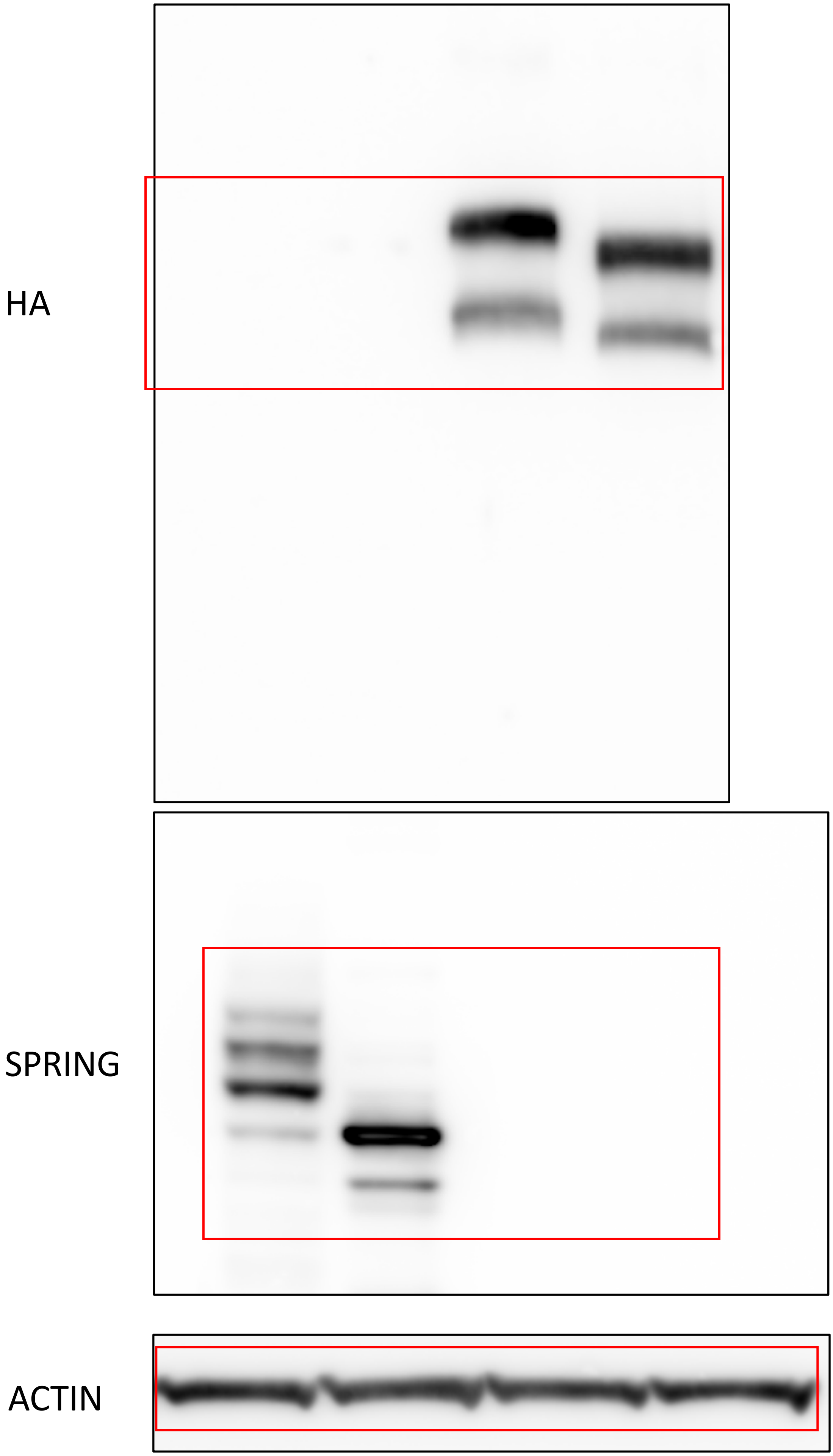

Supplementary Figure 6D

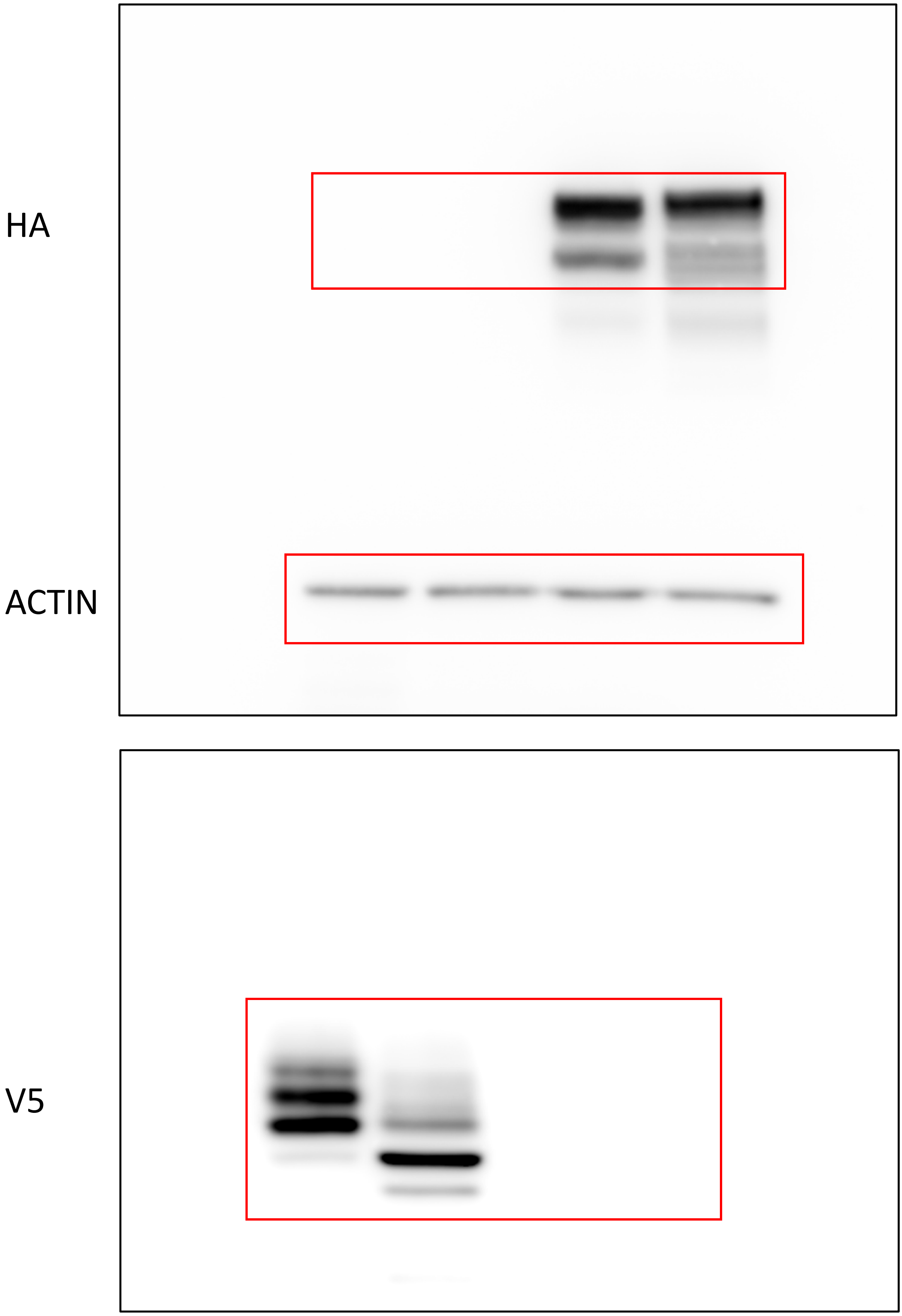

Supplement: Supplementary file 9 — Source Data [file 41467_2020_14811_MOESM9_ESM.zip › Source Data.pdf]
